# The South African Rugby Injury and Illness Surveillance and Prevention Project (SARIISPP): The Currie Cup Premiership Competition Injury Surveillance Report, 2024

**DOI:** 10.17159/2078-516X/2026/v38i1a24534

**Published:** 2026-01-15

**Figure:**
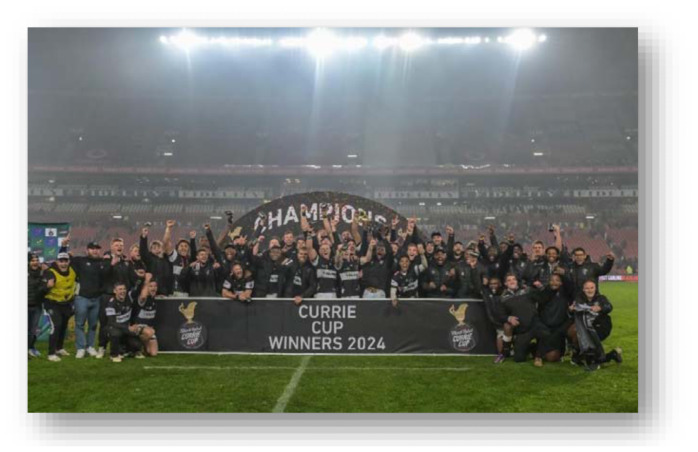

## Executive Summary

As part of the South African Rugby Injury and Illness Surveillance and Prevention Project (SARIISPP), the medical doctors and medical support staff of the respective teams recorded the injury data for the annual Currie Cup 2024 Premiership Division Competition (‘Currie Cup’). SARIISPP has been collecting and analysing these data annually since 2014 for the Currie Cup tournament. For the initial data collection period, 2014–2022, only seven teams were in this tournament. However, since 2023, the Currie Cup competition was expanded to eight participating teams. All eight teams must record the injuries that occur in each match and training session in their team throughout the season. The strength and conditioning coaches also recorded their training session data to calculate training exposure data throughout the season. By combining match exposure data, training exposure data, and injury data, SARIISPP aims to understand the factors contributing to player injury risk.

The analysis reveals injury patterns and facilitates comparisons across various years, tournaments, teams, and the scientific literature, both locally and internationally. Based on the evidence, areas of concern can be identified, and appropriate changes in the game, tournament structure, or medical support services can be considered. Furthermore, when the evidence supports such actions, injury-specific interventions can be developed and implemented.

This report uses injury severity and injury rate as key metrics for analysis. It is important to note that even if teams maintain a low injury rate, high-severity injuries can still impose a significant injury burden on the team. Such injuries result in a considerable number of training and match days lost due to injury. This emphasises the necessity of collecting data on injury severity and injury rates.

The injury rates are expressed as the mean (± 95% confidence intervals) per 1000 player exposure hours. A total of 131 Time-Loss injuries were recorded during match play. The injury rate of Time-Loss injuries for the Currie Cup 2024 was 76 (63 to 89) injuries per 1000 player hours. This is lower than the international meta-analysis injury rate of 91 (77 to 106) injuries per 1000 player hours at a comparative level of play; however, the rates are not significantly different [1]. The injury rate in 2024 falls within the expected limits of season-to-season variation for the Currie Cup. This equates to 1.5 injuries per team per match or 3 team injuries for every 2 matches played, with an average injury burden per team of 2134 days lost per 1000 player hours.

The HOLLYWOODbets Sharks, who subsequently won the tournament, had the highest injury rate for Time-Loss injuries throughout the Currie Cup 2024 tournament. This is the second consecutive year that this outcome was observed. The HOLLYWOODbets Sharks however recorded the second lowest injury severity during the tournament, an outcome which again, was similar to the 2023 Currie Cup competition winners. Toyota Free State Cheetahs had a significantly lower injury rate in 2024 than their 2014–2023 tournament average. The Novavit Griffons had the highest average severity of 67 days absent per injury, whereas the Suzuki Griquas had the lowest average severity of 14 days absent per injury. The average severity of Time-Loss injuries in the 2024 tournament was 28 days, similar to the 27 days reported in the international meta-analysis [1]. The median injury severity of all Time-Loss injuries was 21 days, with 25% of injuries lasting 12 days or less and 25% lasting 43 days or more due to injury.

The most common injury types observed during the 2024 Currie Cup tournament were ligament sprain injuries, with central nervous system injuries and muscle (rupture/strain/tear) injuries ranked second and third, respectively.

The head, ankle, and knee were the most injured body locations in 2024 in that order. Knee injuries decreased, and head injuries remained relatively similar to 2023. Ankle injuries increased by seven percent from 2023. Concussions decreased slightly in 2024 to an injury incidence of 17 (11 to 24) concussions per 1000 player hours. *Ball Carrier* injuries, 22 (15 to 28) injuries per 1000 player hours, accounted for the most injuries in the 2024 Currie Cup tournament, followed by *Open play - contact* and *Tackling*, at 16 (10 to 22) injuries per 1000 player hours and 15 (9 to 12) injuries per 1000 player hours respectively.

In summary, *injury severity*, not just *injury rate*, is crucial for team success. This outcome has been observed for the second consecutive year. In 2024, there was a shift towards more severe injuries. Lastly, the prevalent injury location was the *Head*, followed by the *Ankle*. *Ball Carrier* injuries were the most prevalent in 2024, followed by *Open Play – contact* injuries and *Tackling* injuries.

**Figure:**
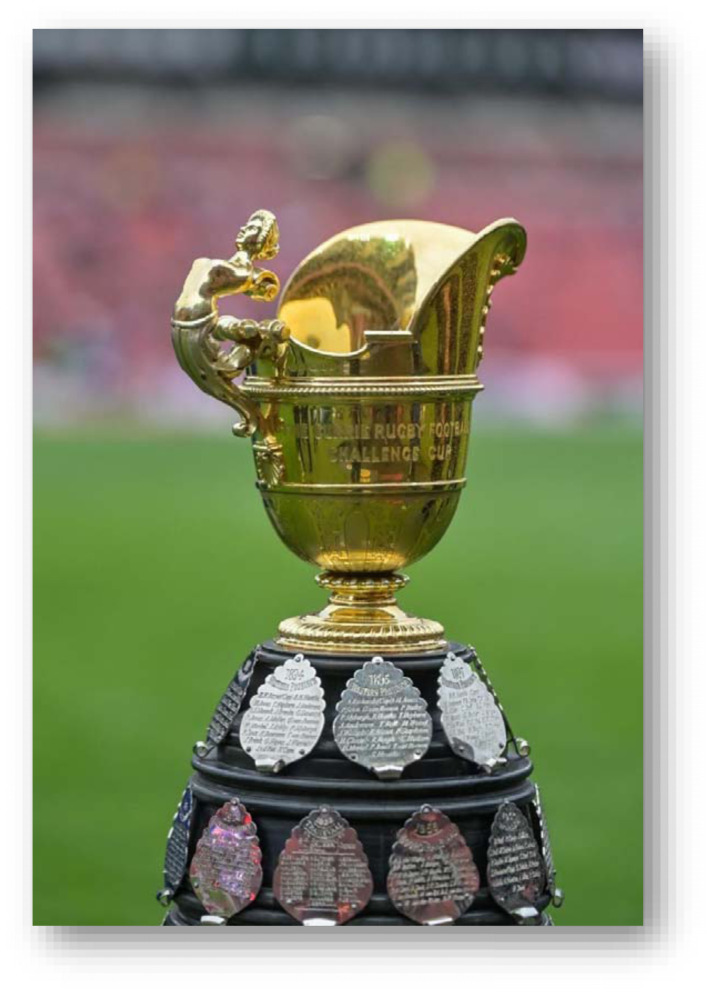

## Introduction

In 2014, the South African Rugby Union (SA Rugby) introduced a standardised injury surveillance format for the Currie Cup Premiership Division Competition as part of the South African Rugby Injury and Illness Surveillance and Prevention Project (SARIISPP). This format required the team’s medical doctor or medical support staff to record all relevant injury data from matches and training sessions using the standardised BokSmart injury surveillance data capture format. The definitions and reporting format used in this system are aligned with the consensus statement on injury definitions and data collection procedures for studies of injuries in rugby union [2] and with the IOC consensus statement for injury recording in sport [3].

Injury surveillance is a crucial step in preventing injuries. Specifically, it is important for developing injury prevention strategies and assessing their efficacy and effectiveness after implementation. By capturing injury surveillance data in a standardised format, it becomes possible to compare injury rates between teams participating in the same tournament, track tournament injuries over consecutive years, and compare findings with other rugby injury surveillance studies. This standardised approach enables comprehensive analysis and enhances the ability to make well-informed, evidence-based decisions regarding injury patterns and potential prevention strategies.

Reports on rugby tournament injuries typically present the incidence of injuries as a rate, calculated by dividing the total number of injuries by the total amount of time at risk. In this paper, the standard format is to report the number of injuries per 1000 player exposure hours. Match exposure hours are determined by multiplying the number of matches played by the number of players involved (30) and the match duration (80 minutes); for team-specific match-related exposure, 15 players would be used. Training exposure hours are computed by multiplying the average number of players at training by the average time spent training each week. These totals are then summed to obtain the overall training exposure hours during the competition period. This report provides standardised injury rates to facilitate comparisons with other studies. Every effort has been made to present these rates on a ‘per team’ and ‘per match’ basis for easier and more practical interpretation.

Since 2016, the Currie Cup medical doctors and medical support staff have been asked to record the injured players’ physical return to play date, thereby calculating the actual severity of the injury. Injury burden is a combination of the injury rate and severity and is expressed as the number of days absent from training and matches per 1000 player hours. Throughout this report, only actual, rather than predicted severity is used for analysis.

The report includes data from the 2014 and 2015 seasons only in sections reporting injury numbers and incidence. The sections reporting injury severity and burden begin with the 2016 season, the first time actual severity data were collected.

In the Currie Cup 2020/21 seasonal report, the South African Rugby Injury and Illness Surveillance and Prevention Project (SARIISPP) began capturing Time-Loss training injuries and training exposure data. This addition enables SARIISPP to gain a more comprehensive understanding of injury data by combining match exposure, training exposure, and injury data.

An inherent bias with most injury surveillance studies is that the teams’ medical doctors or medical support staff are responsible for entering their team’s injury data. As no audit process is done to verify these data, in many cases, the accuracy of the data depends on the compliance of the medical doctors or medical support staff. This potential limitation is present in most injury surveillance studies. SARIISPP had a project coordinator who communicated regularly with the medical doctors or support staff to minimise this potential limitation. This ensured that data capturing was up to date.

The Currie Cup 2024 semi-finals were contested between the Vodacom Blue Bulls, HOLLYWOODbets Sharks, Fidelity ADT Lions, and Toyota Free State Cheetahs. The final was between the Fidelity ADT Lions and HOLLYWOODbets Sharks, with the HOLLYWOODbets Sharks eventually winning the tournament.

**Figure:**
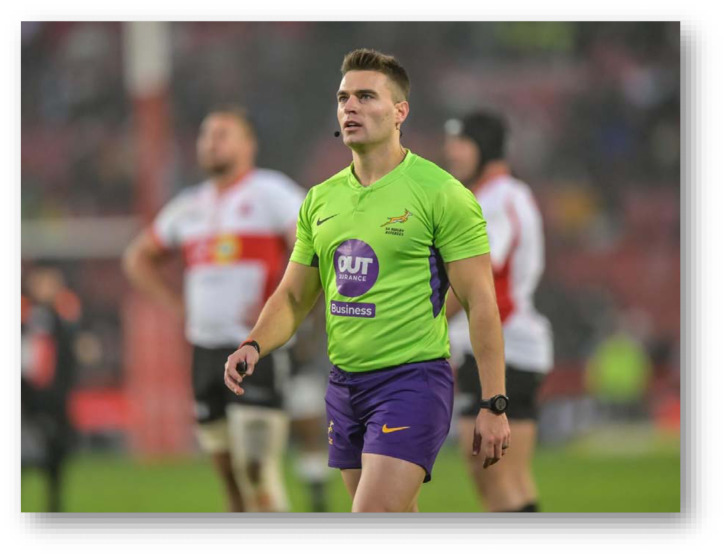

## Definitions

All definitions are originally based on the 2007 consensus statement for injury reporting in rugby union [2] and have since been realigned with the latest International Olympic Committee (IOC) consensus statement for methods of recording and reporting epidemiological data on injury and illness in sport [3].

### MEDICAL ATTENTION INJURY

All injuries seen by the teams’ medical doctors or medical support staff were classified as Medical Attention injuries. These injuries are defined by the 2007 statement as an “*injury that results in a player receiving medical attention”* [2], and by the IOC statement as *“a health problem that results in an athlete receiving medical attention”* [3].

### TIME-LOSS INJURY

Medical Attention injuries were further categorised as Time-Loss injuries, where appropriate, and defined by the 2007 statement as “*an injury that results in a player being unable to take a full part in future rugby training or match play*” [2]. The IOC definition is *“a health problem that results in a player being unable to complete the current or future training session or competition”* [3]. For clarity, this means an injury sustained by a rugby union player during a match or training session that prevented or would have prevented the player from taking full part in all rugby training activities and/or match play for more than 1 day following the day of injury, irrespective of whether match or training sessions were scheduled [4].

### INJURY RATE

This report defines an injury rate as the number of injuries expressed per 1000 player exposure hours. This method of expressing injury rate has been used in previous years’ reports of the Currie Cup Premiership tournament and other international literature, making comparisons easy. Moreover, the injury rate is expressed as a mean with 95% confidence intervals. A 95% confidence interval around a mean value indicates a 95% chance (i.e., very high chance) that the true value falls within this range. In this report, we present the 95% confidence intervals assuming a normal distribution of the data and use the approach of examining the overlap of the confidence intervals to determine whether the injury incidences are significantly different; if the range of confidence interval values of two comparisons do not overlap, there is a strong chance (95%) that their injury rates are different from each other. We have opted for this method because it is easy to use, conservative, and less likely to produce false positive results [5].

### MEDIAN (INTERQUARTILE RANGE)

When numbers are ordered from the lowest to the highest, the median separates the higher half of the values from the lower half. Simply put, it is the middle value of a list of ranked numbers. The interquartile range (IQR) describes the spread of the data. When rank-ordered data are divided into quartiles, the first and third quartiles represent the values under which 25% and 75% of the data points fall, respectively. For example, consider a team with a median injury severity of 32 days (IQR 7 to 40). This means that when the teams’ injury severities are ranked in order, the mid-point or median of the injury severities is 32 days. Also, 25% of their injuries result in 7 or fewer days absent from training and matches, and 25% of their injuries result in 40 days or more absent from training and matches.

### NEW, SUBSEQUENT AND RECURRENT INJURIES

In 2024, in the Currie Cup Premiership Division Competition, a ‘*New Injury’* was defined as when a player sustained his first injury. Any injury the *same* player sustained after this initial injury was defined as a *‘Subsequent Injury’*.

According to the IOC statement, any subsequent injury to the same site and of the same type is referred to as a ‘*Recurrence’* if the index injury was fully recovered before reinjury and as an *‘Exacerbation’* if the index injury was not yet fully recovered [3].

To provide more detail on the subsequent injuries for practitioners, we have further categorised the subsequent injuries in this report into one of four groups based on the Orchard Sports Injury and Illness Classification System (OSIICS) [6, 7] classification diagnosis:

- Different site - Different type
- Different site - Same type
- Same site - Different type
- Same site - Same type

According to the 2007 Consensus Statement for rugby, any subsequent injury classified as ‘Same site - Same type’ was a *‘Recurrent injury’ [*2].

### INJURY SEVERITY

The total severity of an injury is defined as *“the number of days that have elapsed from the date of injury to the date of the player’s return to full participation in team training and availability for match selection”* [2,3]. The actual severity of each injury is classified by the severity groupings provided in the 2007 consensus statement: *Slight* (0–1 days lost), *Minimal* (2–3 days lost), *Mild* (4–7 days lost), *Moderate* (8–28 days lost), *Severe* (>28 days lost), *Career ending*, and *Non-fatal catastrophic* [2]. To align with the latest IOC statement, the injuries have been re-grouped to reflect the severity groupings *‘1–7 days’, ‘8–28 days’ and ‘>28 days’* [*3*].

The average severity represents the average number of days lost per injury, calculated by dividing the total number of days lost by the total number of injury events. For example, a team may have a total severity of 550 days absent, accumulated from 22 injuries. The average severity of the team’s injuries would therefore be 550/22, which equals, on average, 25 days absent per injury.

### INJURY BURDEN

The combined injury rate and severity determine injury burden. It is calculated by multiplying the injury rate by the average severity (number of days lost due to injury). It is the number of days absent per 1000 player hours. For example, consider a team with an injury rate of 75 injuries per 1000 player exposure hours and an average severity of 38 days lost per injury. In this case, the injury burden for the team would be calculated as 2850 days absent per 1000 player hours (i.e., 75 × 38 = 2850).

### OPERATIONAL INJURY BURDEN

The operational injury burden is the expected number of days lost due to injury per team for every match played over the tournament or season. The measure extrapolates injury rates and severities over a season and includes the most severe and least severe injuries in its estimation. For example, suppose a team has an operational injury burden of 2 days. In that case, it means that, based on their injury rates and average severity, and within the team, 2 playing or training days are lost due to injury for every match the team plays.

### META-ANALYSIS

A meta-analysis uses statistical methods to combine multiple scientific studies with varying levels of evidence on the same topic. The goal is to determine overall defining patterns and results based on the combined data. As such, it represents the highest level of scientific evidence available. The findings in this report are compared to the data in the most recent meta-analysis, which was published in 2021. The meta-analysis specifically focuses on rugby union injuries at an elite professional level [1].

**Figure:**
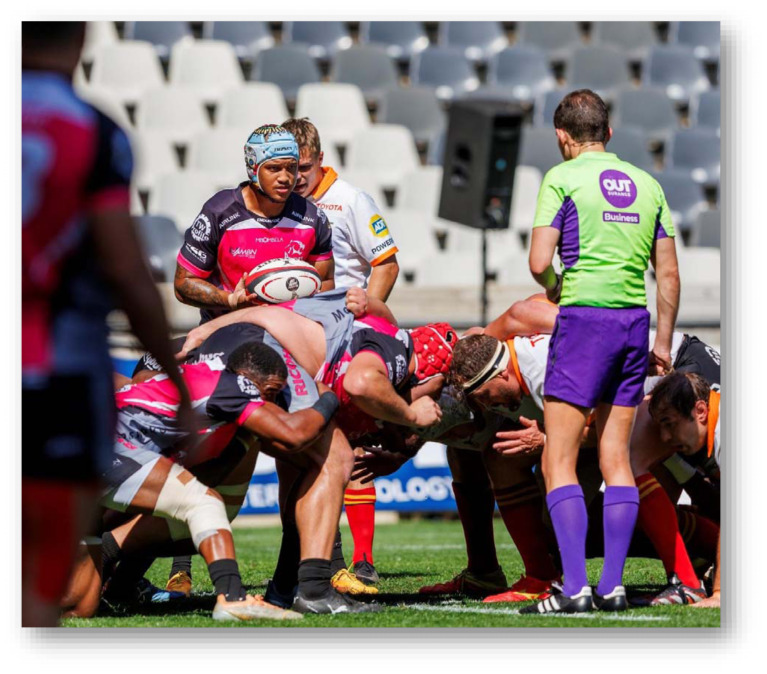

## MATCH INJURIES

### Injured players

During the Currie Cup 2024, 111 players sustained 131 Time-Loss injuries. Due to squad changes over the tournament duration for various reasons, a total number of 369 different players were physically exposed to injury at some point while playing rugby matches as part of the Currie Cup 2024 tournament. However, for analysis and exposure calculation purposes, we assumed 184 players were available to play rugby on match days in the tournament (8 teams × 23 players per match-day squad). Sixty percent (60%) of the 184 available match-day players sustained a match injury during the tournament (Figure 1a). The proportion of players who sustained one Time-Loss injury increased by ten percent (10%) in 2024. The proportion of players who sustained two Time-Loss injuries decreased from 21% in 2023 to 11% in 2024. Furthermore, the proportion of players who experienced 3 injuries remained relatively unchanged in 2024 (Figure 1b). Over the last two years, no players sustained more than 3 injuries. Only the absolute number of Time-Loss injuries was analysed further in this report (n = 131), regardless of the number of players who sustained them.

### Overall Injury Rate

Only Time-Loss injuries have been analysed in this report because these injuries are more comparable between different teams, tournaments and with the published scientific literature [1]. As mentioned, Time-Loss injuries resulted in players missing a match or training session.

**Figure 2:**
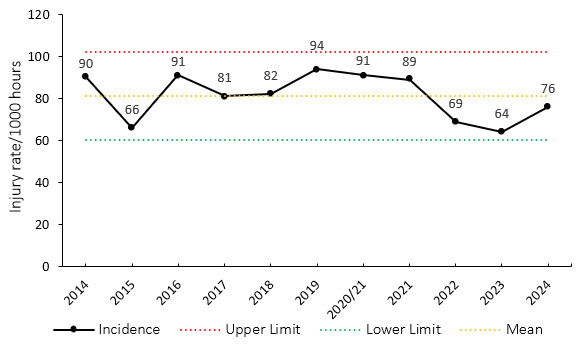
Injury incidence of Time-Loss match injuries over the surveillance period with mean ± standard deviations shown. The red dotted line represents the mean plus standard deviation. The green dotted line represents the mean minus standard deviation. 2020/21 – was a hybrid tournament structure that started in 2020 and continued into the beginning of the 2021 season due to COVID-19 lockdown interruptions.

The overall match injury incidence for the Currie Cup 2024 was 76 (63 to 89) injuries per 1000 player exposure hours. The 2024 Currie Cup tournament’s injury rate is lower than, however, it is not significantly different to the international meta-analysis injury rate of 91 (77 to 106) injuries per 1000 player hours [1]. It falls within the season-to-season variation for the Currie Cup, based on the last 10 years’ collective data (Figure 2). An incidence of 76 injuries per 1000 player hours equates to 1.5 injuries per team per match or roughly 3 team-injuries for every 2 matches played.

**Figure 3:**
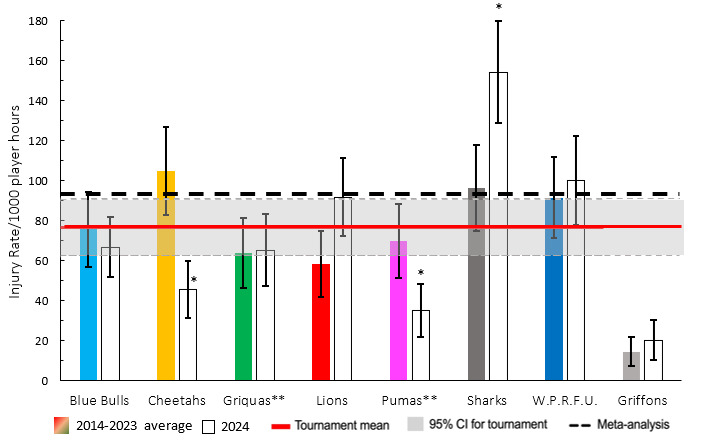
Injury incidence rates for Time-Loss injuries experienced by each team in the Currie Cup 2024 compared to their 2014–2023 average. (*) indicates that a team’s 2024 injury rate significantly differs from its 2014–2023 average injury rate. The whisker lines for each bar represent the 95% Confidence Interval. (**) Average injury rates for Pumas from 2015 – 2023 and Griquas for 2015, 2016, 2018–2023.

When comparing each team’s 2014–2023 average tournament injury incidence to their 2024 season’s injury incidence data, the Toyota Free State Cheetahs and Airlink Pumas experienced significantly lower injury incidence rates in 2024 (Figure 3). The HOLLYWOODbets Sharks, in 2024, experienced a significantly higher injury rate.

Overall, the combined average injury incidence of 77 (63 to 91) injuries per 1000 player hours for all the teams over the last 11 years is similar to the international meta-analysis summary of 91 (77 to 106) injuries per 1000 player hours [1].

**Figure:**
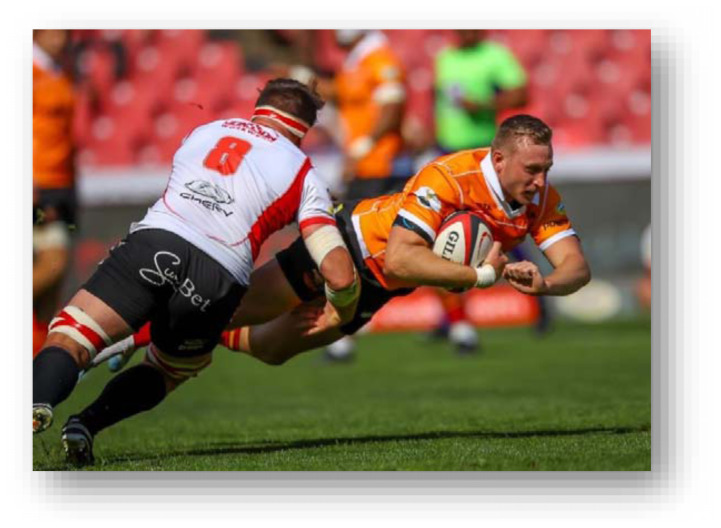

**Figure:**
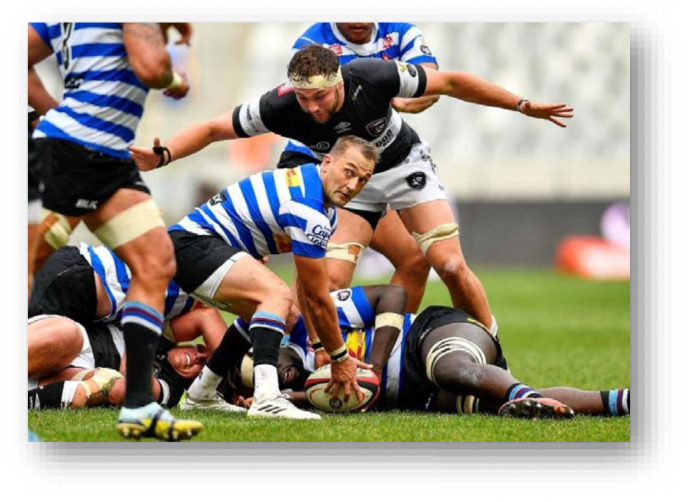

### Injury incidence over the season

**Figure 4:**
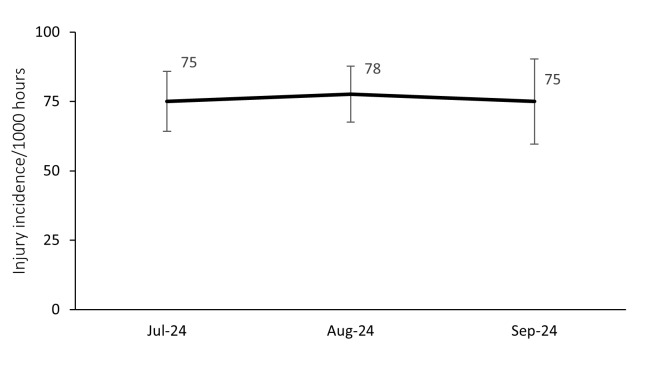
Match injury incidence per month of the 2024 Currie Cup season. The whiskers for each point represent the 95% Confidence Intervals.

The 2024 Currie Cup tournament consisted of one round of matches from July to September. This was different to the 2022 and 2023 competitions, which both featured double-rounds and were played in the first half of the year. Table 1 shows the different tournament formats from 2014 to 2024. When exploring the Time-Loss injury incidence during the 2024 Currie Cup tournament, there were no significant differences between the tournament months in the 2024 season (Figure 4).

### Overall Severity

**Figure 5:**
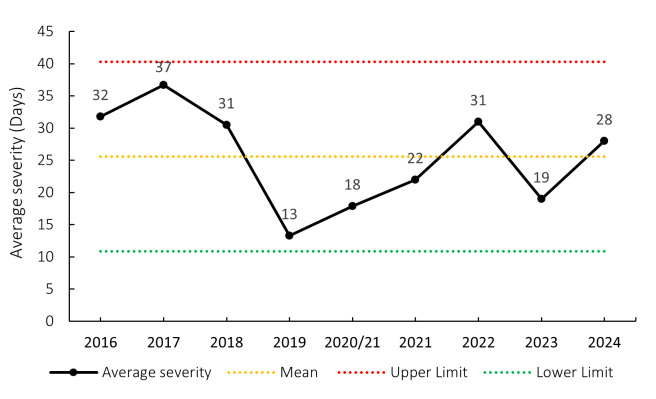
Mean severity of Time-Loss match injuries over the surveillance period, with mean ± standard deviations shown. The red dotted line represents the mean plus standard deviation. The green dotted line represents the mean minus standard deviation. 2020/21 – was a hybrid tournament structure that started in 2020 and continued into the beginning of the 2021 season due to COVID-19 lockdown interruptions.

The average severity of match injuries for the Currie Cup 2024 was 28 days, which was within the expected season-to-season variation (Figure 5). Match injury severity increased consistently from 2019 to 2022 and decreased in 2023. The severity increased again in 2024. The median severity in 2024 was 21 days (IQR 12 to 43). This means that the halfway mark of the injury severities was 21 days, with 25% of all Time-Loss injuries lasting 12 days or less and 25% lasting 43 days or longer.

When the medical doctors or medical support staff clinically assessed the injured player, they recorded the injury time from the date that the injury occurred as the starting date. Similarly, the return to play date was recorded when the player returned to full participation in team training and availability for match selection. The injury severity was determined from the difference between these two dates.

These data are grouped to align with the latest IOC statement. The severity groupings include *‘1–7 days’, ‘8–28 days’ and ‘>28 days’* [*3*].

**Figure 6:**
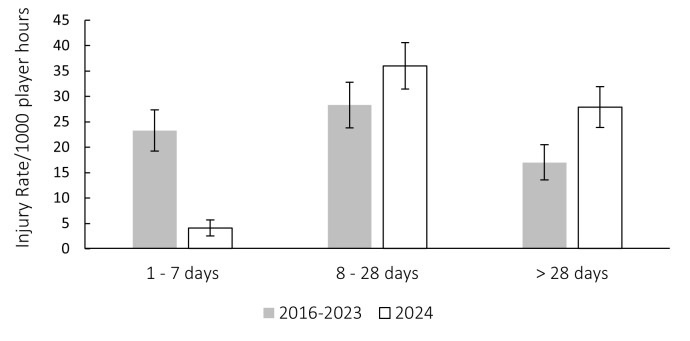
The actual severity category injury rates for the Currie Cup 2024 compared to their average rates for 2016–2023. The whiskers for each bar represent the 95% Confidence Intervals. (*) indicates that the injury incidence of actual severity category ‘1–7 days’ lost is significantly lower than its category average for 2016–2023. (**) indicates that the injury incidence of actual severity category ‘>28 days’ lost is significantly higher than its category average for 2016–2023.

Figure 6 compares the injury severity rates for the Currie Cup 2024 tournament to the average injury severity rates of the 2016–2023 tournaments. Injury rates in the severity category of ‘*1–7 days’* were significantly lower in 2024 compared to their 2016–2023 average, while ‘>28 days’ were significantly higher in 2024 compared to their 2016–2023 average. This suggests that there were significantly more ‘*severe*’ injuries in 2024 than in the 2016–2023 surveillance period before that (Figure 6). This is worth monitoring.

**Figure 7:**
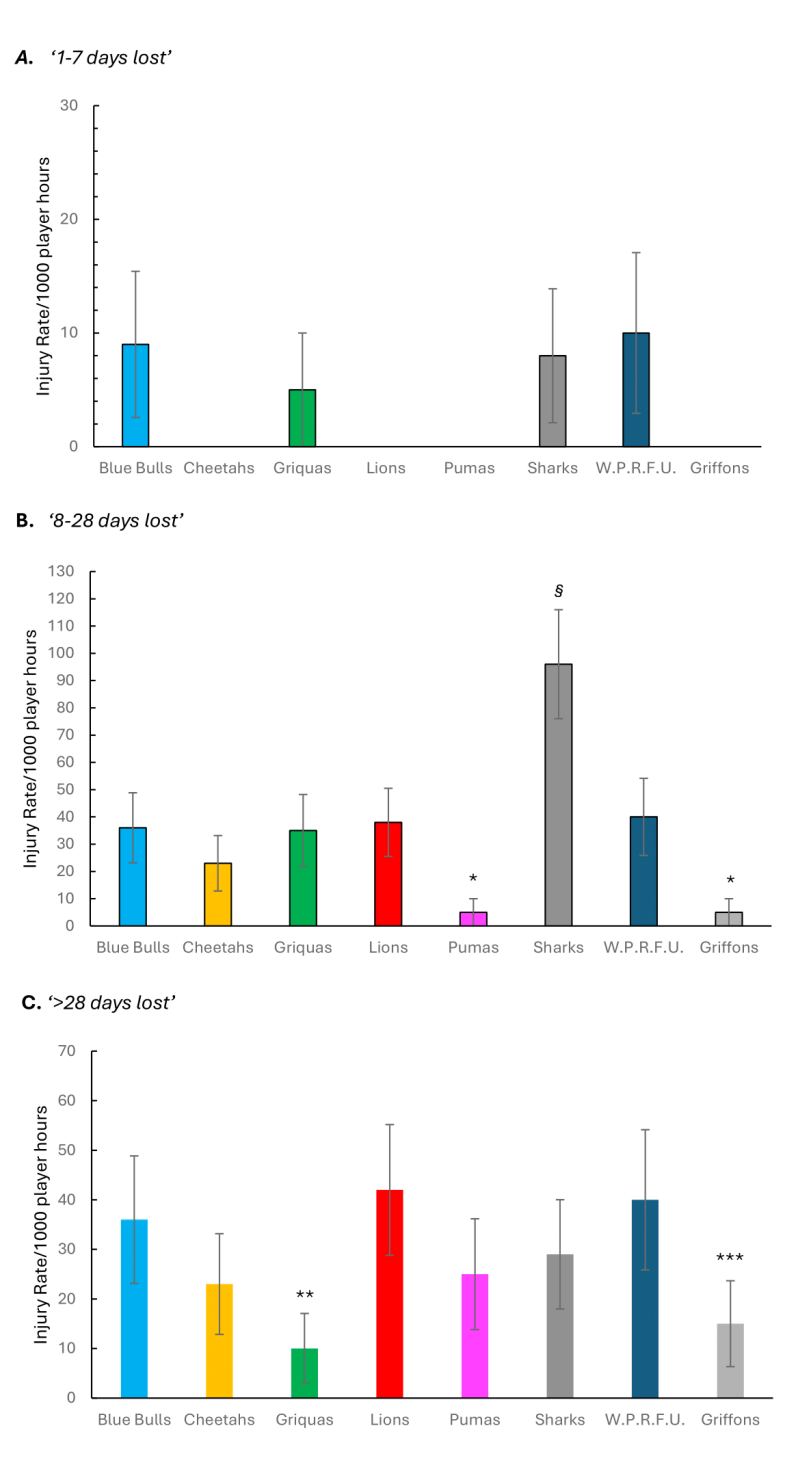
The actual severity category injury rates for the Currie Cup 2024 for each team. The whiskers for each bar represent the 95% Confidence Intervals. A. Injury rates in the severity category of ‘1–7 days lost’. B. Injury rates in the severity category of ‘8–28 days lost’. C. Injury rates in the severity category of ‘>28 days lost’. (*) indicates that the injury incidences of the actual severity category ‘8–28 days lost’ for the Pumas and Griffons were significantly lower than the rest of the teams’ injury rates. (§) indicates that the injury incidence of the actual severity category ‘8–28 days lost’ for the Sharks were significantly higher than the rest of the teams’ injury rates. (**) indicates that the injury incidence of the Griquas’ actual severity category ‘>28 days lost’ was significantly lower than the Blue Bulls, Lions, Sharks and W.P.R.F.U. injury rates. (***) indicates that the injury incidence of the Griffons’ actual severity category ‘>28 days lost’ was significantly lower than the Lions and W.P.R.F.U. injury rates.

Figure 7 shows the actual severity category injury rates in the Currie Cup 2024 tournament for each team. Injury rates of the Griquas in the severity category *‘>28 days lost*’, was significantly lower than the injury rates of the Blue Bulls, Lions, Sharks and W.P.R.F.U. The Griffons injury rates was significantly lower than the Lions and W.P.R.F.U. injury rates in the *‘>28 days lost’* severity category.

**Figure:**
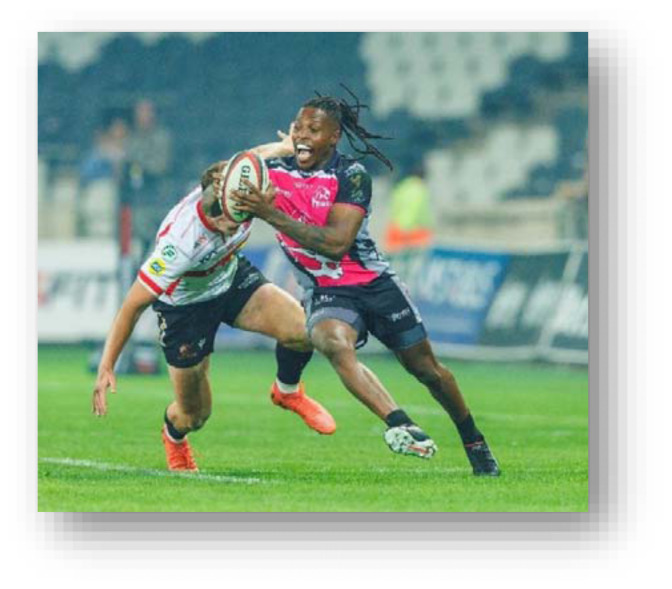

Table 2 describes the relationships between incidence, actual severity, and injury burden of each teams’ Time-Loss injuries for the Currie Cup 2024. The HOLLYWOODbets Sharks have been used as a worked example to explain Table 2. The HOLLYWOODbets Sharks sustained 3.1 injuries per match, meaning that for every 0.3 matches played, they sustained one injury. The HOLLYWOODbets Sharks lost 939 training and match days due to injury. This equates to an average of 25 training and match days lost for every injury sustained. The burden of the team’s injuries equates to 3913 days lost per 1000 player hours. Translating this to an operational burden per match shows that the HOLLYWOODbets Sharks, within their player group, effectively lost 78.3 days due to injury per match played over the season. The median injury severity for the HOLLYWOODbets Sharks was 17 days (IQR 12 to 26). This means that when severities of the HOLLYWOODbets Sharks Time-Loss injuries were rank ordered, the midpoint of the severities was 17 days off from rugby, with 25% of their injuries lasting equal to or less than 12 days off, and 25% of their injuries lasting equal to or longer than 26 days off.

**Figure:**
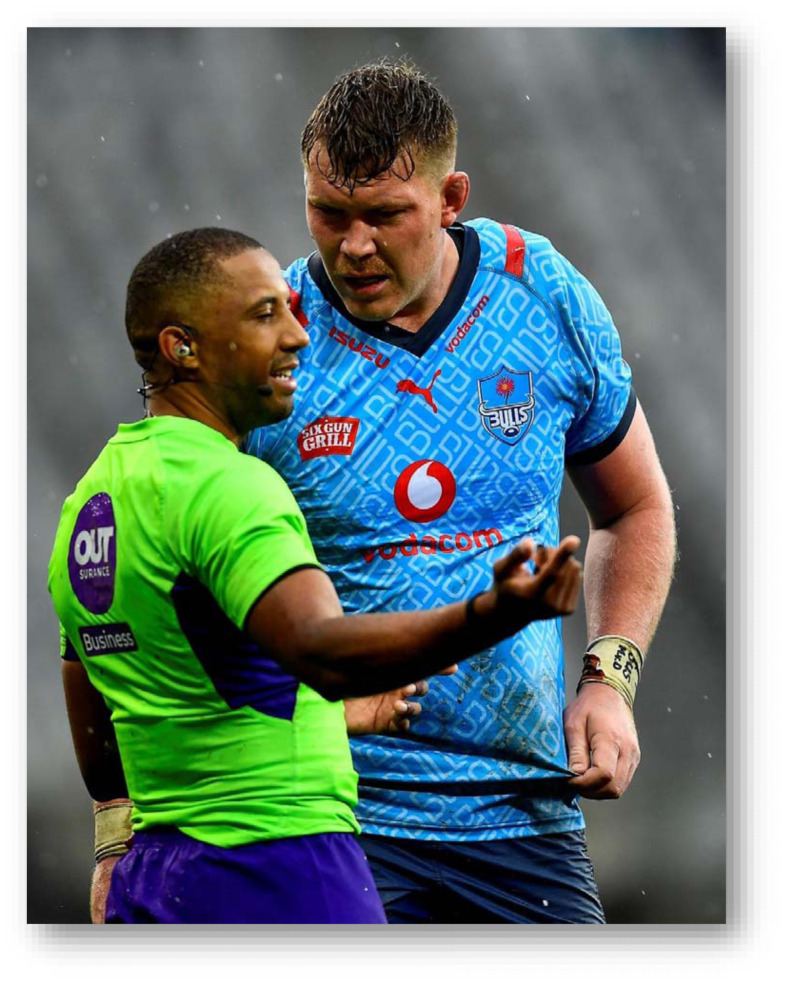

**Figure 8:**
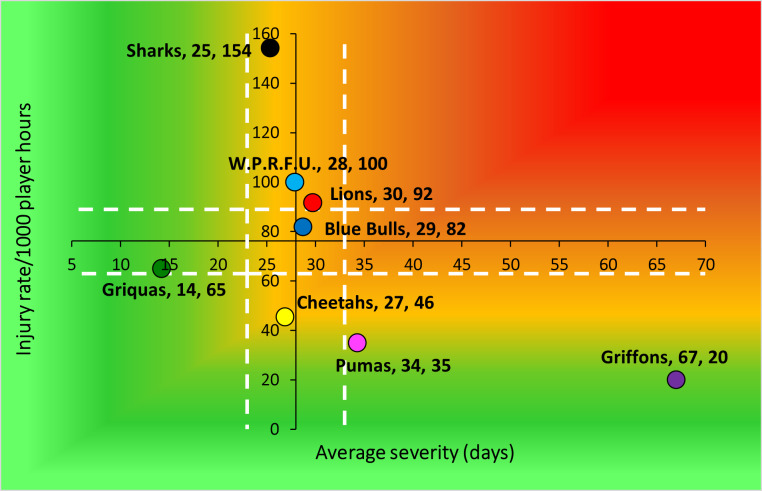
Injury rate plotted against the average severity of Time-Loss injuries for each participating team in the Currie Cup 2024. The Y-axis Average Injury Rate intercept is expressed as the tournament average (the horizontal white dotted lines represent 95% Confidence Intervals), and the X-axis Average Severity intercept is expressed as the average of all the individual injury severities in the tournament (the vertical white dotted lines represent 95% Confidence Intervals).

The HOLLYWOODbets Sharks had the highest Time-Loss injury rate, followed by the DHL Western Province and Fidelity ADT Lions. In contrast, the Suzuki Griquas had the lowest injury severity, which resulted in the lowest injury burden per team. Novavit Griffons had the lowest injury incidence. However, their average severity was high, resulting in an injury burden of 1330 days lost per 1000 player hours (Table 2; Figure 8). Previous studies have shown that teams with lower injury rates were more successful in the Currie Cup competition [8, 9]. However, as per the last few years, this was not the case in the 2024 Currie Cup. It has also been shown that injury burden, rather than injury rates alone, needs to be considered for success in this tournament [10]. Teams that fall in the green zone (below average and 95%CI), will generally not be impacted as much by their injury burden, regardless of whether their injury rate or average severity is relatively high. When the combination of rate and severity moves into the orange (close to average) and/or red zone (above average and 95% CI), the impact on team performance and player availability becomes more problematic. In the 2024 Currie Cup the Fidelity ADT Lions was the team closest to the red zone. Although HOLLYWOODbets Sharks had the highest total injury burden, their average severity was low, preventing them from approaching the red zone. Fidelity ADT Lions had a high injury incidence coupled with a higher injury severity, which resulted in them approaching the red zone.

All the data in this report are aligned with the 2019 IOC consensus statement [3] and are further presented as such to compare against previous season reports and the international meta-analysis [1]. Table 3 presents the Currie Cup 2024 injury data in the format recommended by the 2019 IOC consensus statement. This table provides an overview of the Tissue and Pathology types of injuries sustained during the 2024 season. This format is used throughout the report.

### New, Subsequent and Recurrent Injuries

During the Currie Cup 2024, the overall injury incidence for *New injuries* was 74 (61 to 87) injuries per 1000 player hours.

**Figure 9:**
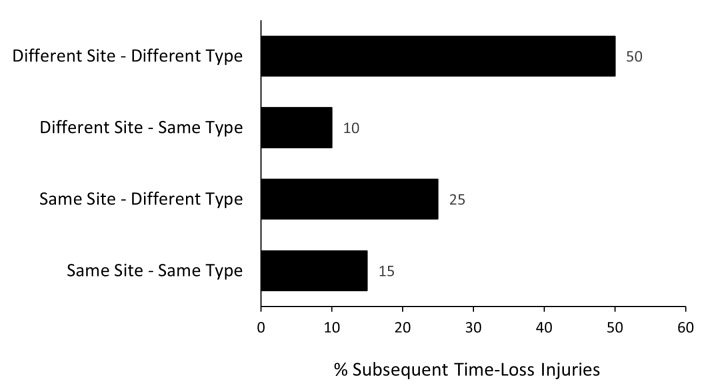
Classification of subsequent injuries for the Currie Cup 2024. Data expressed as a % of subsequent Time-Loss injuries.

Ninety-five players experienced one injury during the Currie Cup 2024 season (86% of all injured players). Fifty percent (50%) of subsequent injuries in the 16 players who sustained multiple injury events during the season (Figure 1a and Figure 9), occurred at a different anatomical site and were of a different type compared to the initial index injury. ‘*Different site – different type*’, ‘*different site – same type*’ and ‘*same site – different type*’ are classified as subsequent new injuries. Figure 9 shows the percentage breakdown of subsequent Time-Loss injuries into these categories.

**Figure:**
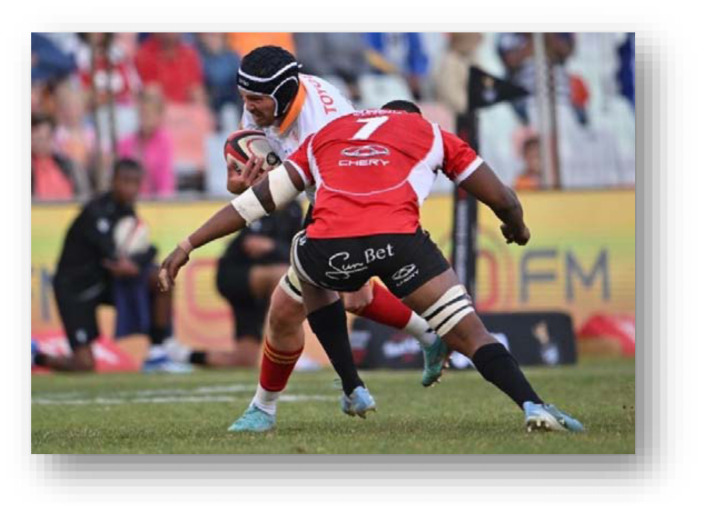

A subsequent *recurrent* injury was any subsequent injury classified as ‘*same site – same type*’, which refers to the same location and same tissue type involved as the original index injury. Only three subsequent recurrent injuries occurred in the Currie Cup 2024.

The injury incidence in 2024 for subsequent recurrent injuries was 2 (0 to 4) injuries per 1000 player hours, which is higher than the 2023 tournament’s injury incidence of 1 (0 to 2) per 1000 player hours. However, due to the small number of these injuries, it was not significantly different.

The proportion of new injuries decreased, and subsequent recurrent injuries increased slightly compared to the Currie Cup 2023 tournament (Table 4).

**Figure:**
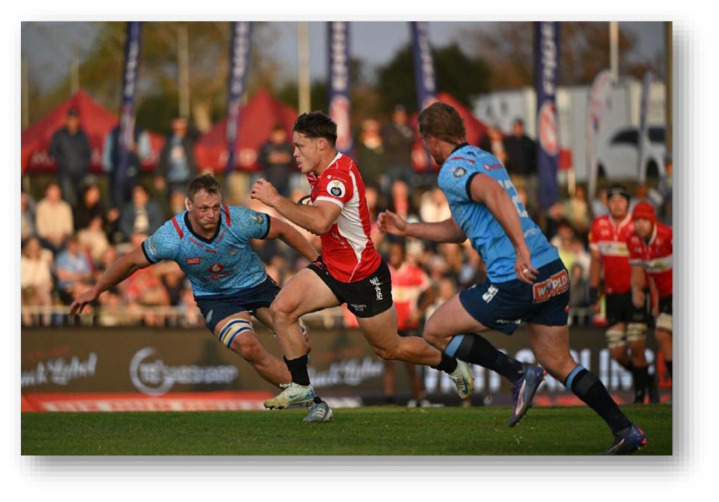

### Player injuries per positions

**Figure 10:**
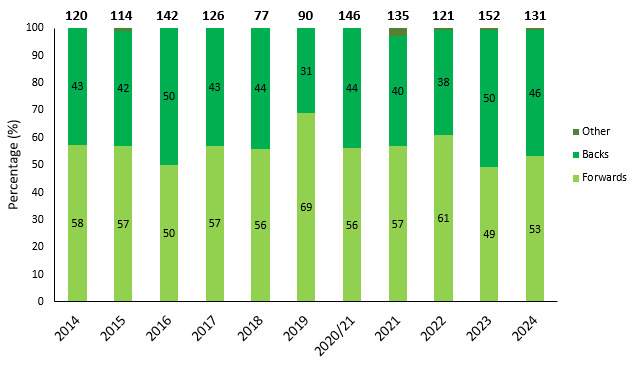
Proportion of injuries occurring to forwards and backs and total number of injuries from 2014 to 2024. (The number above each bar represents the total number of injuries for that year). 2020/21 – was a hybrid tournament structure that started in 2020 and continued into the beginning of the 2021 season due to COVID-19 lockdown interruptions.

Figure 10 shows the total number and proportion of injuries occurring to forwards and backs per year. The proportion of injuries occurring to forwards and backs has been undulating throughout the past eleven years. In the last two years, the proportions of injuries occurring to forwards and backs have been similar (Figure 10).

**Figure:**
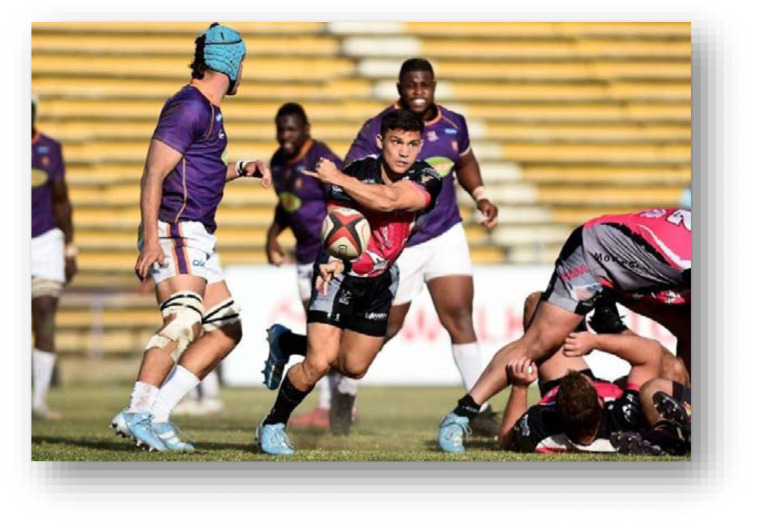

### Injury Type

Overall, Sprain Ligament injuries were the most common Time-Loss injuries recorded during the Currie Cup 2024 (22%), followed by Central Nervous System injuries (21%).

The median severity for Sprain Ligament injuries was 44 days, with 25% of injuries resulting in 21 or fewer days absent from training and matches and 25% resulting in 56 or more days absent (Table 5). The average severity was 43 days absent.

**Figure 11:**
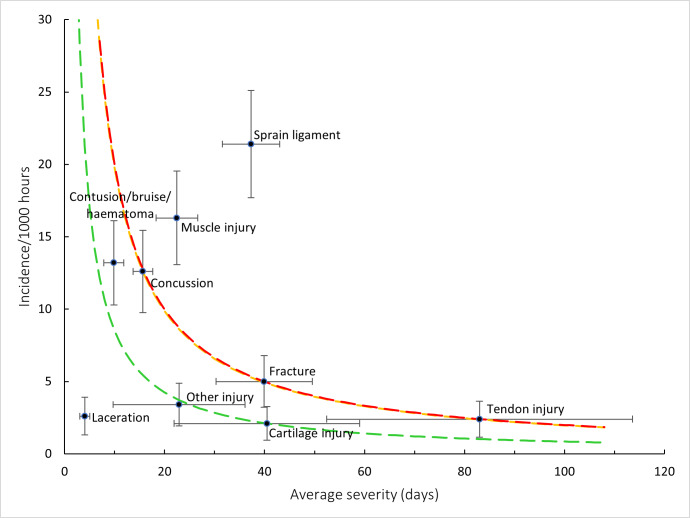
Injury burden as a function of injury type for the seasons 2016 – 2024. The y-axis represents incidence (number of injuries per 1000 hours), and the x-axis represents the average severity (days absent) per injury type. Green line: values to the left and below represent those under the 25^th^ burden percentile; these are low impact injuries. Orange line: values to the left and below represent those under the 50^th^ burden percentile; these include the low-medium impact injuries. Red line: values to the left and below represent those under the 75^th^ burden percentile; these include the medium-high impact injuries. Values to the right and above the red line are the highest impact injuries and affect players and teams the most.

Figure 11 shows the injury burden for the period 2016–2024. Ligament sprain, followed by muscle injury, were the two injury types with the highest burden when data were combined for the 2016–2024 Currie Cup tournaments. These injury types have the highest combination of injury incidence and average injury severity. Consistent with previous reports, these two injury types continue to dominate across the different teams.

The most common Time-Loss injuries during the Currie Cup 2024 tournament were joint (non-bone)/ligament injuries (comprised of dislocation/subluxation and sprain/ligament injuries) recorded at 25 (18 to 33) injuries per 1000 player hours. The average severity for joint (non-bone)/ligament injuries was 42 (33 to 50) days.

Following joint (non-bone)/ligament injuries, central nervous system injuries were the next most common injuries. Central nervous system injuries had an injury incidence of 17 (11 to 24) injuries per 1000 player hours. The average severity of central nervous system injuries in the Currie Cup 2024 was 17 (14 to 20) days. The injury rate for muscle/tendon injuries was 16 (10 to 22) injuries per 1000 player hours. The average severity for muscle/tendon injuries was 37 (19 to 56) days.

### Injury Diagnosis [6,7]

The most common Orchard Sports Injury Classification System (OSIICS) diagnosis ^[6]^ in the Currie Cup 2024 was Concussion (OSIICS code = HN1) followed by Hamstring Strain (TM1) (Table 6).

**Figure:**
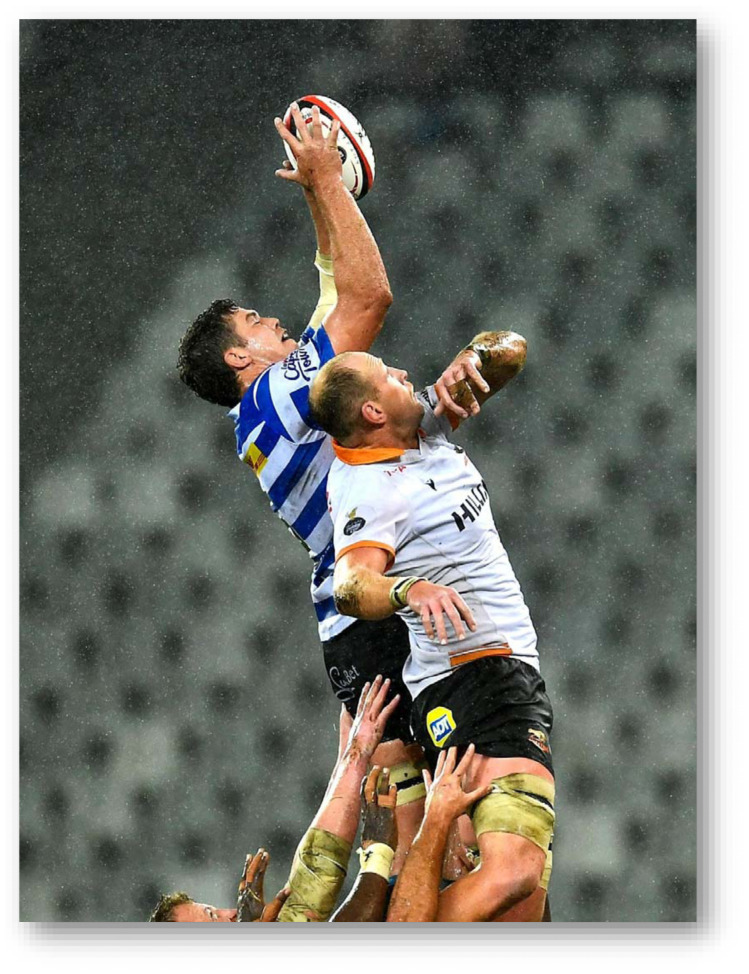

### Concussions

**Figure 12:**
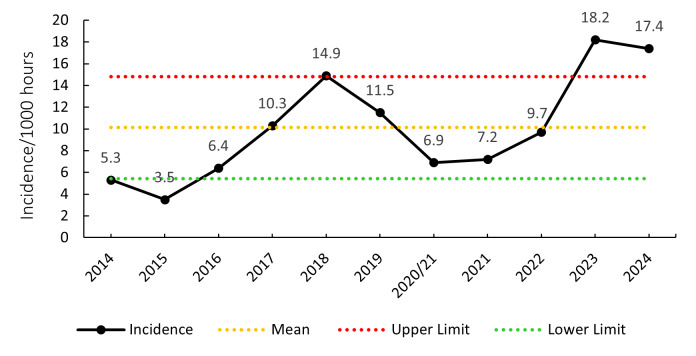
Incidence of concussion over the surveillance period with mean ± standard deviations shown. The red dotted line represents the mean plus standard deviation. The green dotted line represents the mean minus standard deviation. 2020/21 – was a hybrid tournament structure that started in 2020 and continued into the beginning of the 2021 season due to COVID-19 lockdown interruptions.

Concussions contributed to 30 injuries in the Currie Cup 2024 (23%). Concussion incidence decreased from 18.2 concussions per 1000 player exposure hours in 2023 to 17.4 concussions per 1000 player hours in the Currie Cup 2024 tournament. In practical terms, this equates to 0.7 concussions per match, one concussion every 1.4 matches, or one can expect 7 concussions for every 10 matches played. The concussion rate in 2024, for the second successive year, falls outside of the historically expected season-to-season variation for the Currie Cup (Figure 12), with an overall grouped tournament average of 10.1 concussions per 1000 player hours over the entire data collection period. The average severity of concussions reported in the 2024 tournament was 17 days with a median severity of 13 days (IQR 12 – 21 days). World Rugby approved the use of the Head Injury Assessment (HIA) protocol for the Currie Cup Tournament. Players’ concussion management and return to play is directed as per the HIA protocol.

- World Rugby HIA protocol: https://www.world.rugby/the-game/player-welfare/medical/concussion/hia-protocol
- World Rugby Concussion Guideline documents: https://www.world.rugby/the-game/player-welfare
- SARU’s Concussion Guideline documents (*When can a player safely return-to-play*): www.boksmart.com/concussion, and on MyBokSmart: https://my.boksmart.com/Documents/BokSmart#ConcussionManagement

**Figure 13:**
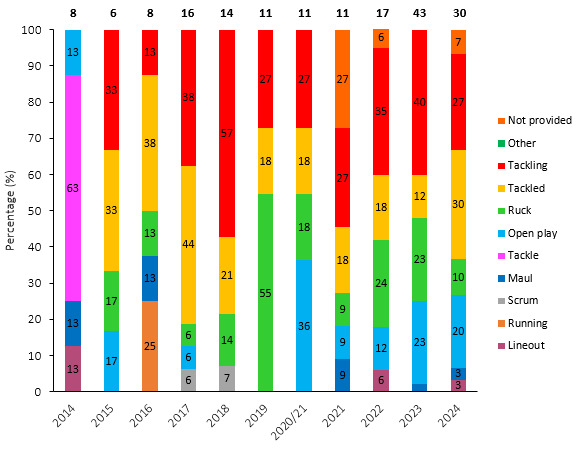
Proportion of concussions caused by the different injury events and total number of concussions from 2014 to 2024. (The number above each bar represents the total number of concussions for that year). 2020/21 – was a hybrid tournament structure that started in 2020 and continued into the beginning of the 2021 season due to COVID-19 lockdown interruptions.

Figure 13 shows the total number of concussions per year, and the proportion of concussions caused by different injury events. The total number of concussions decreased slightly in 2024, because the tournament was only played over one round. The main causes of concussion during the Currie Cup 2024 were *Being Tackled* (30%) and *Tackling* (27%). *Tackler* and *Ruck* concussions both decreased by 13%, but *Ball carrier* concussions went up by 18% in 2024.

**Figure 14:**
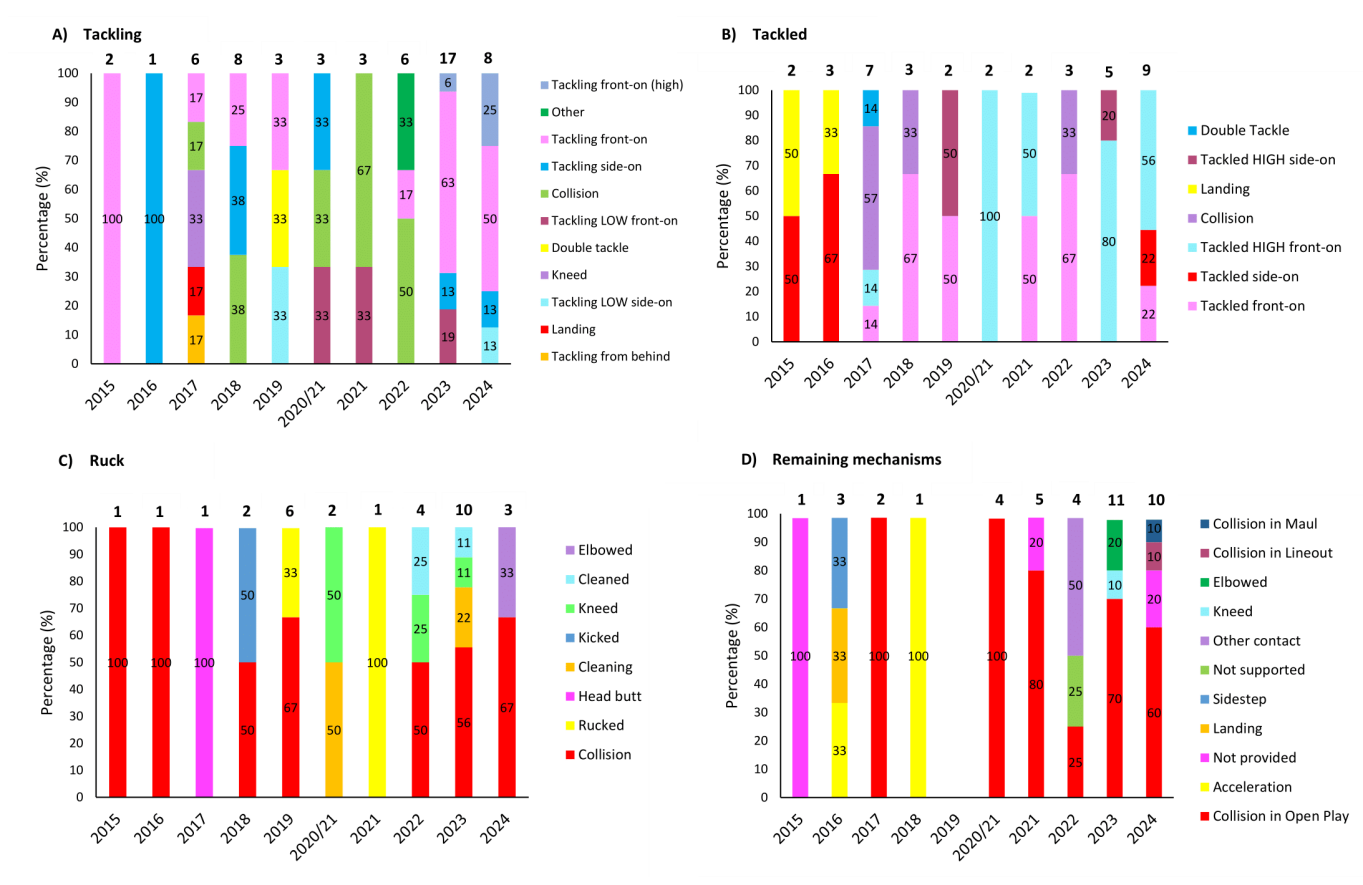
Proportion of concussions caused by A) Tackling, B) Tackled, C) Ruck, and D) Remaining (concussion) mechanisms from 2015 to 2024. The number above each bar represents the total number of concussions for that event for that year. 2020/21 – was a hybrid tournament structure that started in 2020 and continued into the beginning of the 2021 season due to COVID-19 lockdown interruptions.

Figure 14 presents the injury-event mechanisms contributing to concussions in *Tackling, Tackled, Ruck* and the remaining concussion-causing injury events (*Remaining mechanisms*) from 2015 to 2024. Data were only presented from 2015 onwards, as *Tackle-*related data were not captured separately for the *Tackler* and *Ball Carrier* in 2014. *Tackling front-on (regulation)* dominated Tackler concussions in 2024 (50%), being *Tackled front-on high* (56%) in ball carriers, *Collisions* in the Ruck (67%), and *Collisions* in Open Play (60%).

### Injury location

The head was the most frequently injured body location during the Currie Cup 2024 tournament (29%), followed by the ankle (14%). Concussions (n = 30) contributed to the most head injuries. Ligament injuries (n = 16) contributed to the most ankle injuries. Ligament injuries also accounted for the most knee injuries (n = 11) and shoulder injuries (n = 7), whereas muscle strain/spasm injuries (n = 15) contributed to the most thigh injuries.

Head injuries had an average severity of 21 days absent and an injury burden of 462 days lost per 1000 player hours. The median severity of head injuries in the Currie Cup 2024 was 15 (IQR 12 to 27) days absent. Twenty-five percent of head injuries resulted in 12 or fewer days lost from training and matches, and 25% of all head injuries resulted in 27 or more days lost from training and matches (Table 7). The average severity for ankle injuries was 54 days absent, and the injury burden was 594 days lost per 1000 player hours: the highest average severity and burden in 2024. Thigh injuries had the second lowest average severity of 25 days absent and an injury burden of 250 days lost per 1000 player hours. Shoulder injuries recorded an average 54 days absent and 432 days lost, and knee injuries with 30 days absent and 300 days lost per 1000 player hours.

When analysing the changes in incidence of the most injured body locations for the Currie Cup over the past nine seasons (2016–2024), the head consistently ranks top of the list for the past two years and for seven out of the nine years studied. However, the knee decreased from the second most injured body location in 2023 to the second least injured body location in 2024. Additionally, shoulder injuries have moved down to the bottom of the list in 2024 (Table 8).

**Figure 15:**
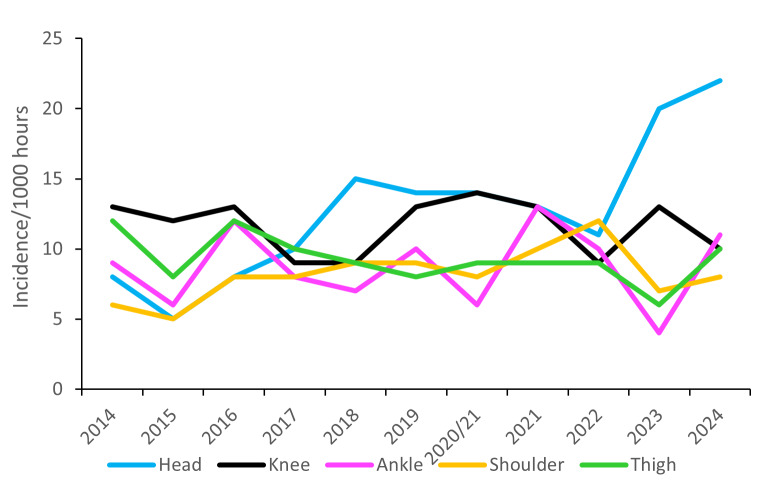
Incidence of the most common injury locations over the surveillance period. 2020/21 – was a hybrid tournament structure that started in 2020 and continued into the beginning of the 2021 season due to COVID-19 lockdown interruptions.

Figure 15 displays the movement of the most common injured body locations over the surveillance period (2014–2024). When examining the injury incidence patterns over the past eleven years, a clear upward trend in head injuries is observed since the 2022 season, reaching the highest recorded rate of head injuries to date in 2024. Shoulder injuries increased gradually between 2015 and 2022 but lowered in 2023 and have remained lowered in 2024. While having decreased sharply in 2023, ankle injuries rebounded in 2024 to a similar level to that experienced in 2022 (Figure 15).

**Figure 16:**
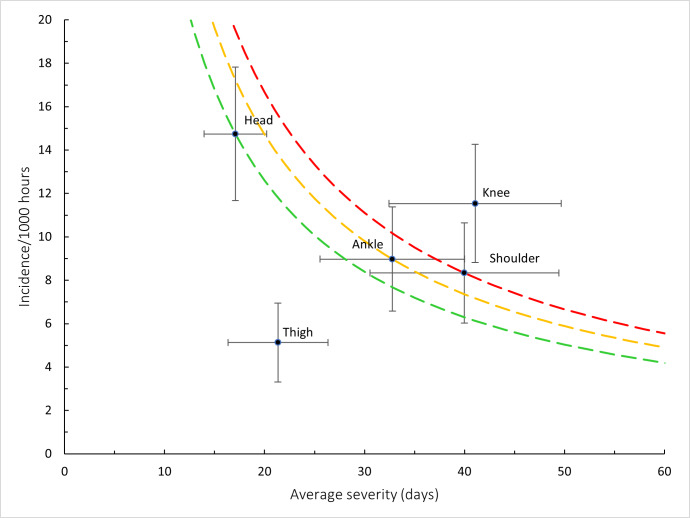
Injury burden as a function of injury location for the seasons 2016 – 2024. The y-axis represents incidence (injuries per 1000 player hours), and the x-axis represents average severity (days absent). Green line: values to the left and below represent those under the 25th burden percentile; these are low impact injuries. Orange line: values to the left and below represent those under the 50th burden percentile; these include the low-medium impact injuries. Red line: values to the left and below represent those under the 75th burden percentile; these include the medium-high impact injuries. Values to the right and above the red line are the highest impact injuries and affect players and teams the most.

Figure 16 combines all the injury location data from 2016 – 2024 and presents the injury burden picture over the past nine years. Injuries to the *Knee* have the highest injury burden for all teams, followed closely by injuries to the *Shoulder*. Both injury locations have a higher combined incidence of injuries and average severity. The *Ankle* followed closely behind these two leading body-locations.

**Figure 17:**
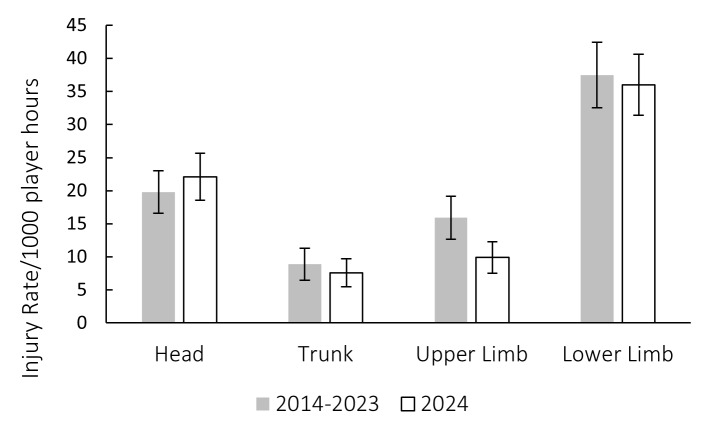
Injury incidence by grouped body location for the Currie Cup 2024 compared to the average 2014–2023 injury rates. The whiskers for each bar represent the 95% Confidence Intervals. * Upper Limb injury rates were significantly lower in 2024 than their 2014–2023 average.

During the Currie Cup, there were no significant differences in grouped body location injury rates between 2024 and their 2014–2023 average injury rates, except for the upper limb body location. Upper limb injury rates were significantly lower in 2024 than their average 2014–2023 injury rates (Figure 17). During the Currie Cup 2024, head and ankle injuries recorded the highest injury rates, with 22 (15 to 29) and 11 (6 to 15) injuries per 1000 player hours, respectively. The head injury rate was similar to that of the international meta-analysis [1] of 17 (14 to 20) injuries per 1000 player hours. The ankle injury rate was also similar to the meta-analysis [1] injury rate of 9 (8 to 11) injuries per 1000 player hours.

**Figure:**
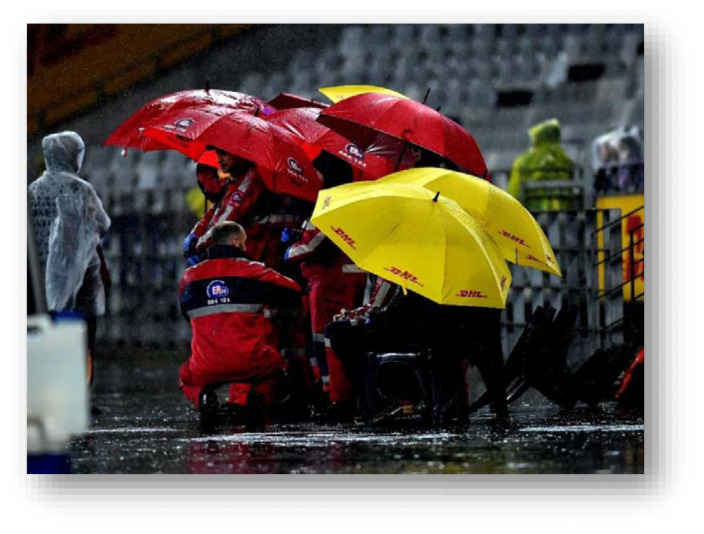

### Injury Event

The *Tackle* event accounted for the most injuries in the Currie Cup 2024 (28%, n = 37), followed by *Open Play – contact* injuries, accounting for 21% (n = 28) (Table 9). When comparing injury rates to the international meta-analysis, *Being Tackled* at 22 (15 to 28) injuries per 1000 player hours was similar to the meta-analysis value of 23 (21 to 25) injuries per 1000 player hours. *Tackling* at 15 (9 to 20) injuries per 1000 player hours was significantly lower than the meta-analysis rate of 23 (21 to 25) injuries per 1000 player hours.

The total severity, median severity and burden of injuries to *Being Tackled* were greater than *Tackling* in 2024. *Open play - running* injury rate during the Currie Cup 2024 season at 8 (3 to 12) per 1000 player hours was similar to the meta-analysis rate of 10 (8 to 13) injuries per 1000 player hours. *Ruck* injury rate at 5 (2 to 9) per 1000 player hours was similar to the meta-analysis injury rate of 9 (7 to 11) per 1000 player hours [1]*. Open play-contact* injury rate at 16 (10 to 22) per 1000 player hours was similar to the *Collision* injury rates in the meta-analysis of 14 (10 to 18) injuries per 1000 player hours.

**Figure:**
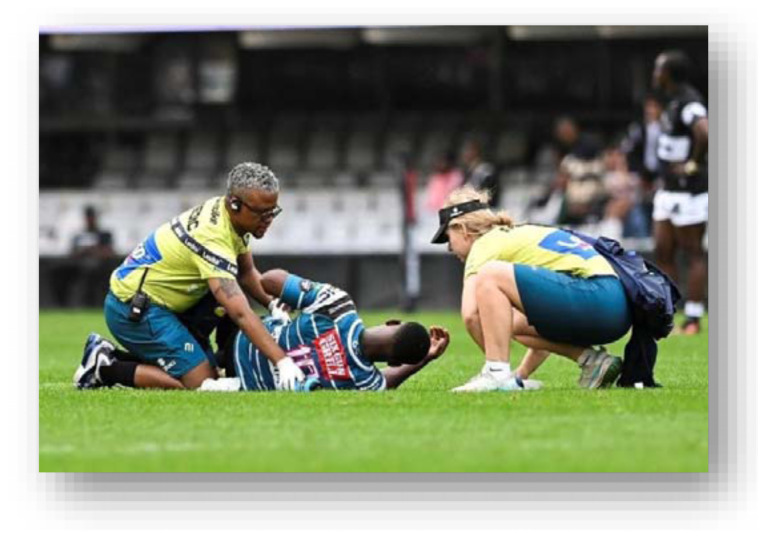

**Figure 18:**
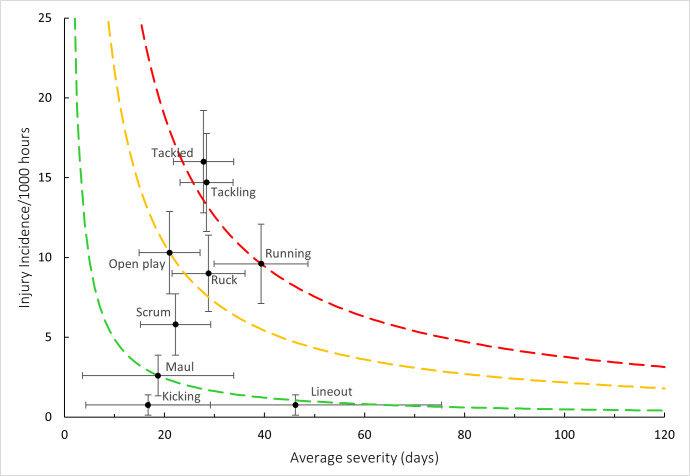
Injury burden as a function of injury event for the seasons 2016 – 2024. The y-axis represents incidence (injuries per 1000 player hours), and the x-axis represents average severity (days absent). Green line: values to the left and below represent those under the 25th burden percentile; these are low impact injuries. Orange line: values to the left and below represent those under the 50th burden percentile; these include the low-medium impact injuries. Red line: values to the left and below represent those under the 75th burden percentile; these include the medium-high impact injuries. Values to the right and above the red line are the highest impact injuries and affect players and teams the most.

Figure 18 combines all the injury events from 2016 – 2024 and presents the injury burden picture over the past nine years. Injuries caused by *Being Tackled* have the highest injury burden for all teams, followed closely by injuries from *Tackling*. Both these injury events have a higher combined injury incidence and average severity. *Running* followed closely behind these two leading injury-causing events.

**Figure 19:**
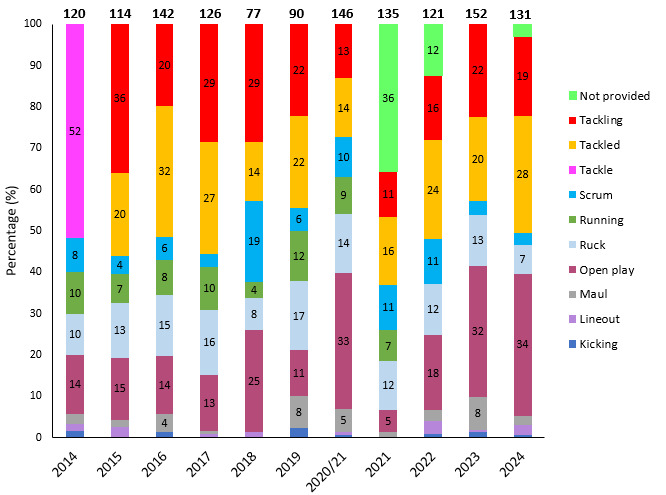
Proportion of injuries caused by the different injury events from 2014 to 2024. (The number above each bar represents that year’s total number of injuries. Tackle data captured separately as tackling and tackled from 2015 onwards). 2020/21 – was a hybrid tournament structure that started in 2020 and continued into the beginning of the 2021 season due to COVID-19 lockdown interruptions.

**Figure 20:**
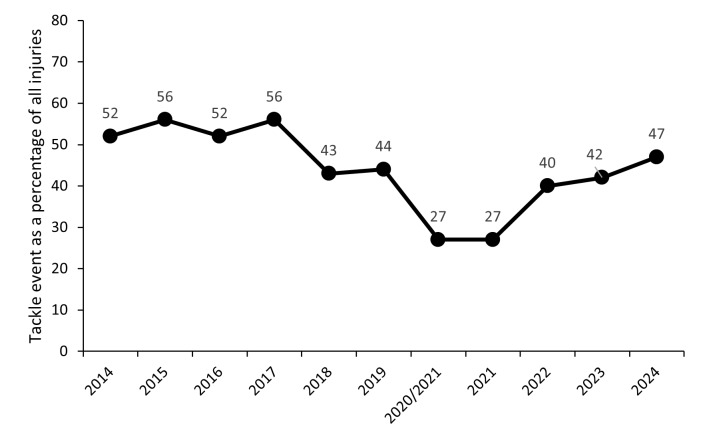
Percentage of all injuries caused by the tackle event from 2014 to 2024. 2020/21 – was a hybrid tournament structure that started in 2020 and continued into the beginning of the 2021 season due to COVID-19 lockdown interruptions.

Figure 19 illustrates the proportion of injuries caused by different rugby events from 2014 to 2024. After dropping in 2018, over the past seven seasons, the rate of injuries caused by tackles has varied, albeit that it has consistently remained lower than the 2014 to 2017 period. After a period of decline between 2018 and 2021, the tackle-related injuries have continued to slowly increase again, largely due to the increasing number of ball carrier injuries and should be monitored moving forward (Figures 19 and 20). The tackle event in the last 11 years has contributed on average to 44% of all injury events in the Currie Cup, and since the roles were split into *Tacklers* and *Ball Carriers* in 2015, 50% of these have been to *Tacklers*, and 50% to *Ball Carriers*.

**Figure 21:**
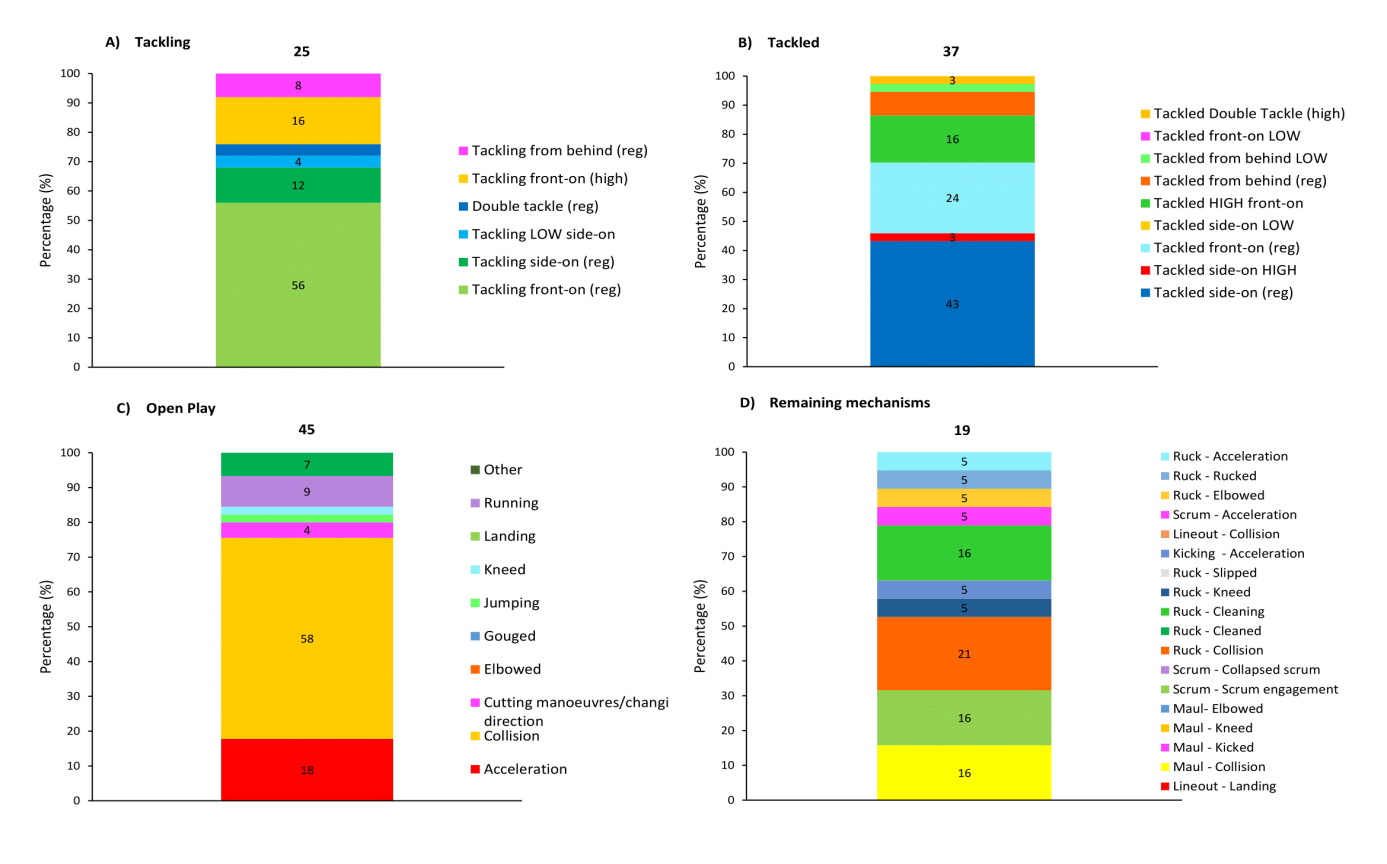
Proportion of injuries per rugby event, caused by A) Tackling, B) Being Tackled, C) during Open Play, and D) the Remaining mechanisms for all other injury causing events in 2024. The number above each bar represents the total number of injuries related to that rugby event for that year. Missing cases = 5.

Figure 21 presents the event mechanisms contributing to injuries in *Tackling, Being Tackled, Open Play* and the *Remaining mechanisms* for all other injury-causing events in 2024. *Tackling front-on (regulation)* dominated Tackler injuries in 2024 (56%), being *Tackled side-on (regulation)* in ball carriers (43%), *Collisions* in Open Play (58%), and *Collisions* in the Ruck (21%).

**Figure:**
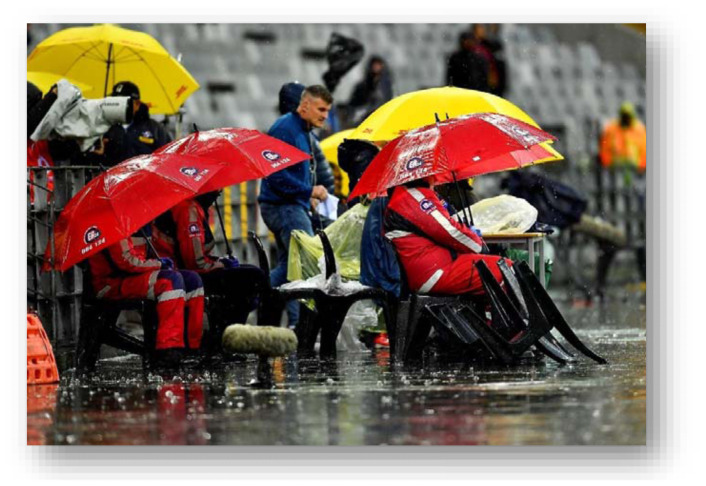

### Venue

**Figure 22:**
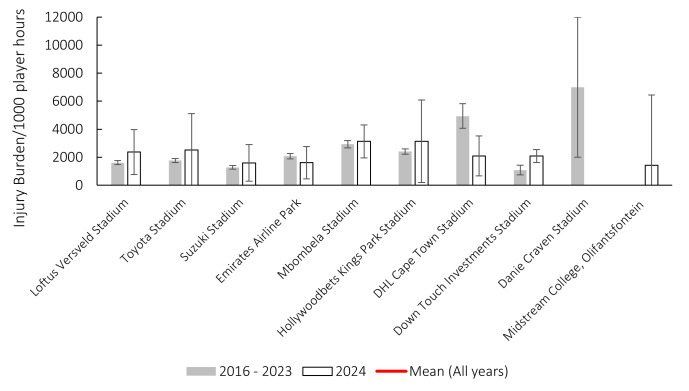
Injury burden/1000 player hours of Time-Loss injuries at the ten utilised stadia in the Currie Cup 2024 in comparison to their averaged 2016–2023 injury burden. * Down Touch Investments Stadium injury burden was significantly higher in 2024 than its 2016–2023 average. ** DHL Cape Town Stadium injury burden was significantly lower in 2024 than its 2016–2023 average. The whiskers for each bar represent the 95% Confidence intervals.

Matches were played at ten different stadia during the tournament. This is the first year that the Midstream College, Olifantsfontein, was used during the Currie Cup tournament. Danie Craven Stadium had no injuries in 2024. DHL Cape Town Stadium’s injury burden was significantly lower in 2024 than its 2016–2023 average, while Down Touch Investments Stadium was significantly higher in 2024 than its 2016–2023 average (Figure 22).

Table 10 shows the ranking of the injury burden of the Stadia from the highest to lowest between 2016 and 2024. Combining the data from the last ten seasons highlights that the DHL Cape Town Stadium, followed by the Mbombela Stadium, recorded the highest overall injury burdens. Suzuki Stadium’s average injury burden was significantly lower than the top five stadia on the list and the grouped average injury burden from 2016–2024. DHL Cape Town Stadium’s average injury burden was significantly higher than five of the nine stadia and the grouped average injury burden between 2016–2024 (Table 10).

**Figure 23:**
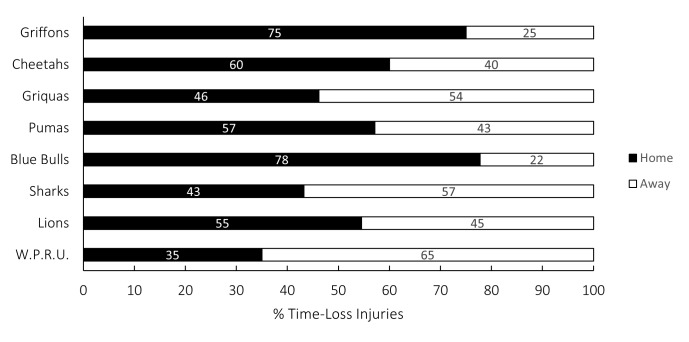
Proportion of injuries sustained playing at home and away venues for the Currie Cup 2024.

Figure 23 presents the proportion of injuries sustained playing at home and away venues in the Currie Cup 2024. When comparing injuries sustained while playing away and at home in the 2024 Currie Cup tournament, the injury rate at home 40 (30 to 49 injuries per 1000 player hours) was similar to that of playing away 37 (28 to 46 injuries per 1000 player hours). The Novavit Griffons, Toyota Free State Cheetahs, Airlink Pumas, Vodacom Blue Bulls and Fidelity ADT Lions, all experienced more injuries when playing at home compared to playing away, while Suzuki Griquas, HOLLYWOODbets Sharks and DHL Western Province Rugby Football Union (W.P.R.F.U.) experienced more injuries while playing away than at home.

## TRAINING INJURIES

**Figure 24:**
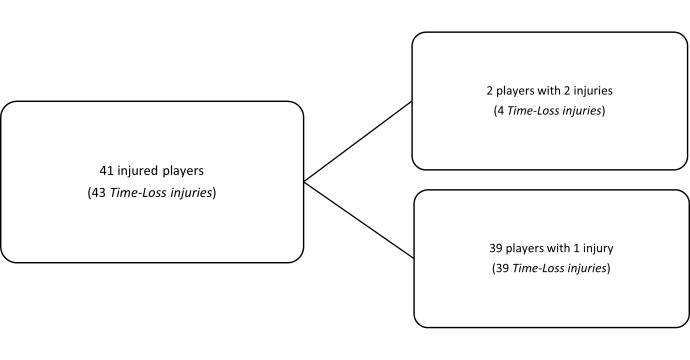
The number of players who experienced Time-Loss injuries in training during the Currie Cup 2024.

**Figure 25:**
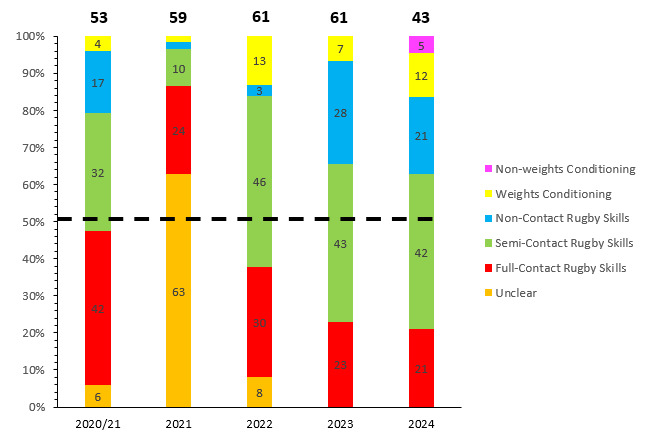
Proportion of Time-Loss training injuries sustained per training activity during the Currie Cup 2020/21–2024 seasons. 2020/21 – was a hybrid tournament structure that started in 2020 and continued into the beginning of the 2021 season due to COVID-19 lockdown interruptions.

Overall, 43 Time-Loss injuries were sustained by 41 players during training in the Currie Cup 2024 season (Figure 24). The Time-Loss injuries resulted in an injury incidence of 2.4 (1.7 to 3.2) injuries per 1000 training hours, which is similar to the meta-analysis injury incidence of 3.0 (1.9 to 4.0) injuries per 1000 training hours [1]. These Time-Loss injuries contributed to 25% of all injuries experienced during the Currie Cup Tournament over the 2024 rugby season (n = 43 training + 131 match = 174 injuries in total). The average severity of training injuries was 28 days, with a median severity (IQR) of 19 (7 to 35) days absent. Figure 25 shows the percentage of training injuries per training activity. Semi-contact rugby skills accounted for the highest percentage of training injuries, which can be expected given the nature of contact and the time spent involved in those activities. Injuries associated with non-contact training and full-contact injuries decreased slightly. Weights- and non-weights conditioning injuries increased slightly in 2024.

**Figure:**
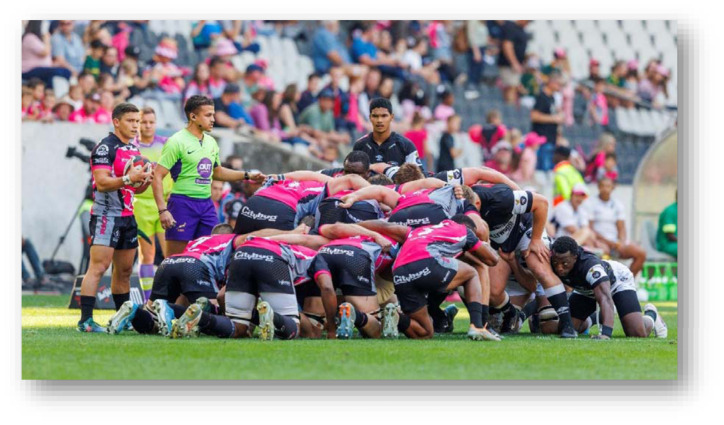

Table 11 presents the training injuries sustained during the Currie Cup 2024. The most common injury type sustained in all training activities was *Muscle Injuries*. *Non-weights conditioning* had the highest average severity of *Muscle Injuries* with 53 days lost, while *Rugby skills (non-contact)* training had the lowest average severity of 18 days.

The *thigh* was the most injured body location in training, accounting for 28% (n = 12) of all Time-Loss training injuries during the Currie Cup 2024, followed by the *lower leg* (14%) (Table 12). *Elbow* training injuries had the highest average and median severities, followed by *shoulder* and *thigh* training injuries. The *Lower body* as a grouped location dominated training injuries at 1.7 (1.1 to 2.3) injuries per 1000 player training exposure hours and experienced the highest injury burden at 47.6 (30.8 to 64.4) injuries per 1000 training hours.

**Figure:**
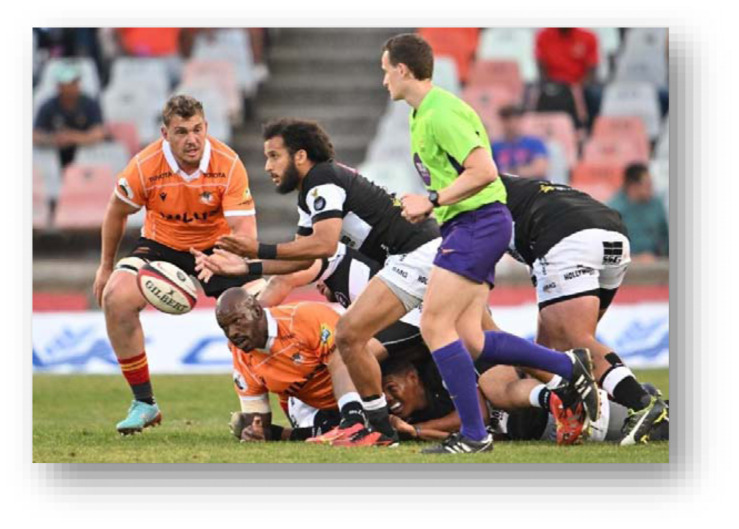

**Figure 26:**
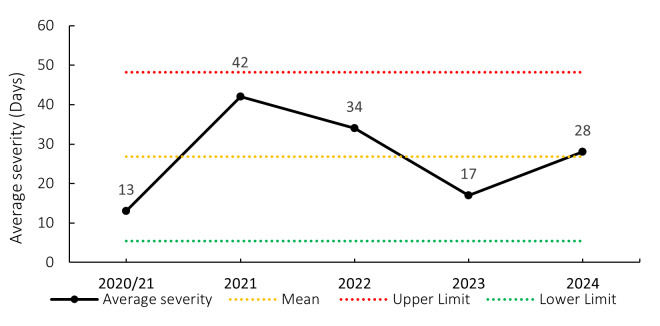
Mean severity of Time-Loss training injuries over the surveillance period with mean ± standard deviations shown. 2020/21 – was a hybrid tournament structure that started in 2020 and continued into the beginning of the 2021 season due to COVID-19 lockdown interruptions.

The average severity of training injuries for the Currie Cup 2024 was 28 days, and this has increased since the 2023 season (Figure 26).

**Figure 27:**
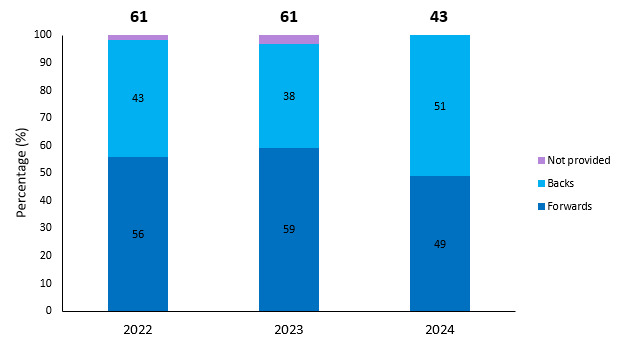
Proportion of injuries occurring to forwards and backs and total number of injuries from 2022 to 2024 during training. (The number above each bar represents the total number of training injuries for that year).

Figure 27 shows the total number and proportion of injuries occurring to forwards and backs per year during training. The proportion of injuries occurring to forwards and backs in 2024 were similar.

**Figure 28:**
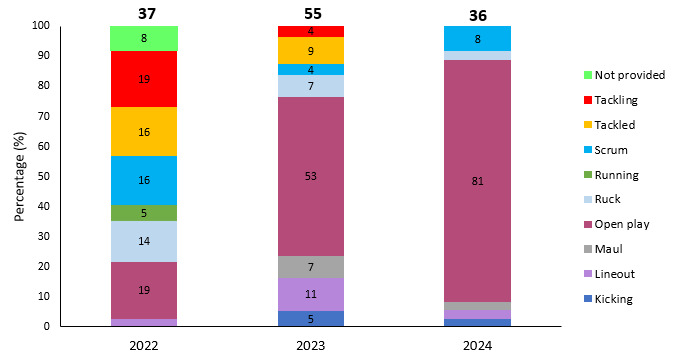
Proportion of injuries caused by the different rugby training events between 2022–2024. (The number above each bar represents the total number of rugby-specific training injuries for that year).

Figure 28 displays the proportion of injuries caused by the different training injury events between 2022–2024. Strength and conditioning-related injury events were removed because they were not rugby-specific events. *Open Play* contributed to the highest proportion of training injuries and experienced a substantial increase in 2024. Training injuries occurring in the tackle event (both *Tackling* and *Tackled* player) decreased in 2023 and again in 2024.

**Figure:**
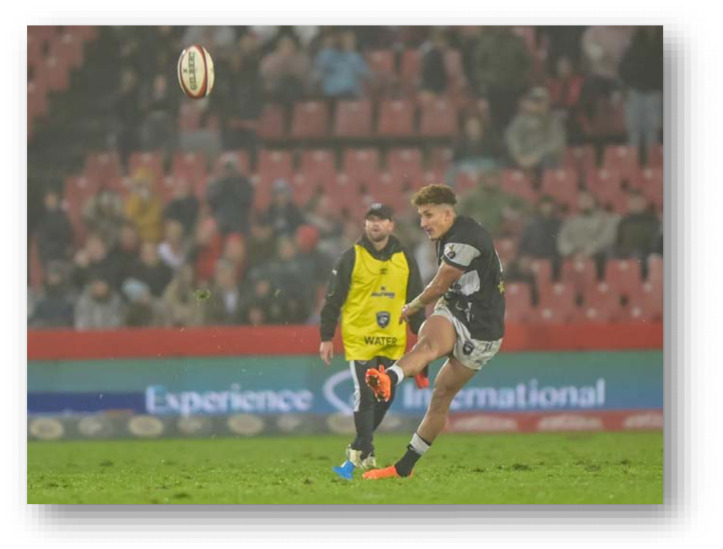

**Figure 29:**
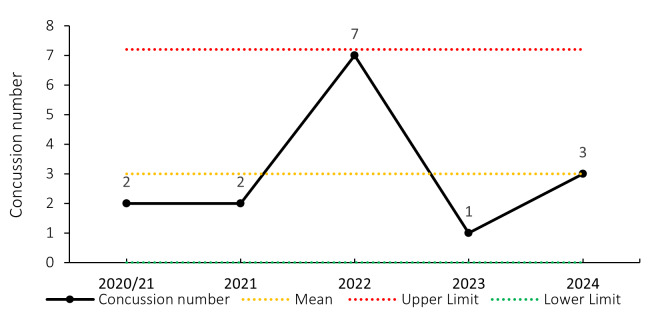
Absolute number of concussions recorded over the training surveillance period. 2020/21 – was a hybrid tournament structure that started in 2020 and continued into the beginning of the 2021 season due to COVID-19 lockdown interruptions.

Overall, concussions contributed to 3 training injuries during the Currie Cup 2024 (7%). This is higher than the 2023 season and the second-highest training concussion number since 2020/21 *(*Figure 29).

**Figure 30:**
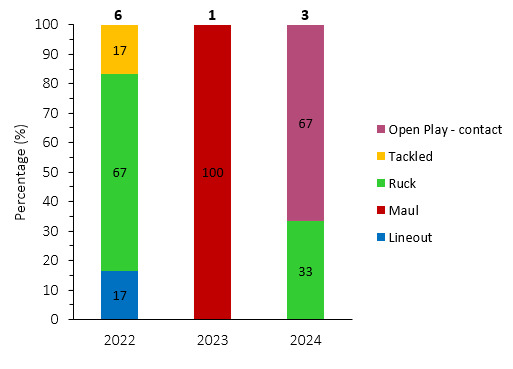
Proportion of concussions caused by the different injury events between 2022–2024 during training (The number above each bar represents the total number of concussions for that year).

In 2024, two training-related concussions occurred during collisions in *Open Play*, and one concussion occurred during a collision in the ruck (Figure 30).

## Take Home Message

1. **Injury Severity and not just Injury Rate, is Crucial for Team Success**: The HOLLYWOODbets Sharks, despite having the highest Time-Loss injury rate during the Currie Cup 2024 tournament, won the competition. This marks the second consecutive year that this outcome was observed. The report highlights that the Sharks recorded the second lowest average injury severity, which suggests that injury severity is a significant factor to consider when assessing a team’s success and performance during a competition.
2. **High-Severity Injuries**: The Currie Cup 2024 tournament experienced a significant increase in the incidence of high-severity injuries (those resulting in more than 28 days lost) compared to the average rates from 2016–2023. Conversely, the incidence of short-duration injuries (1–7 days lost) was significantly lower in 2024. The average severity of match injuries for the tournament in 2024 was 28 days, with a median severity of 21 days.
3. **Sprain Ligament Injuries Were The Most Common Time-Loss Injuries:** During the Currie Cup 2024 (22%), the median severity for Sprain Ligament injuries was 44 days, with 25% of injuries resulting in 21 or fewer days absent from training and matches and 25% resulting in 56 or more days absent.
4. **Prevalent Injury Locations and Events:** The Head was identified as the most frequently injured body location in the Currie Cup 2024, accounting for 29% of injuries, followed by the Ankle (14%) and Knee. Head injuries have consistently ranked as the most common injury for seven out of the nine years studied. Regarding injury events, "Ball Carrier" incidents led to the most injuries (22 per 1000 player hours), followed by "Open play - contact" and "Tackling".

**Figure:**
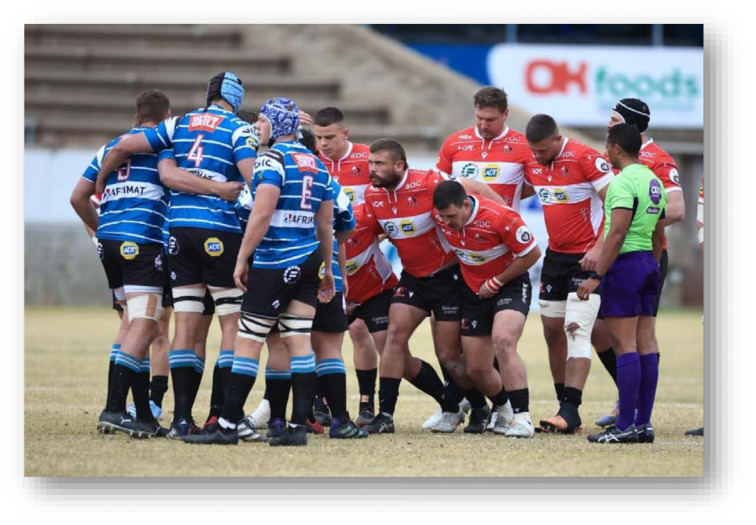

## Figures and Tables

**Figure 1a:**
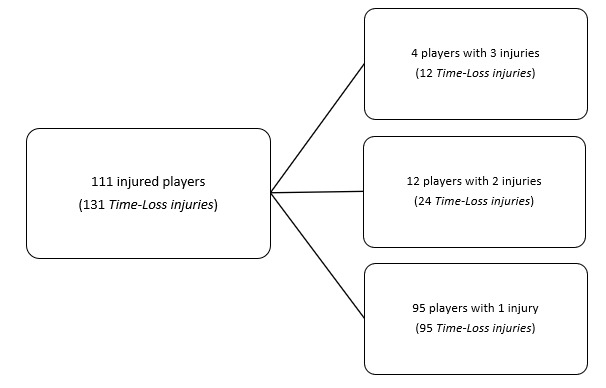
The number of players who experienced Time-Loss injuries during the Currie Cup 2024.

**Figure 1b:**
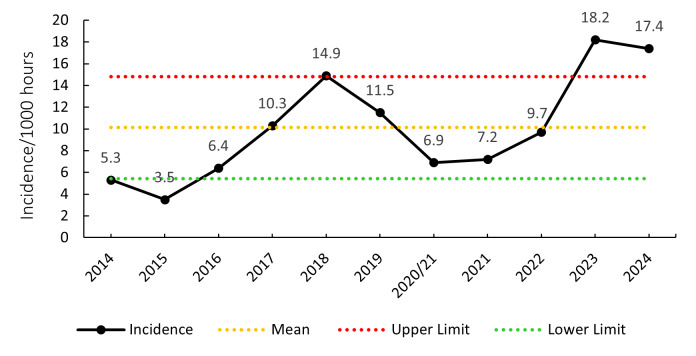
Proportion of injured players experiencing one to five Time-Loss injuries in the Currie Cup tournaments from 2014–2024. 2020/21 – was a hybrid tournament structure that started in 2020 and continued into the beginning of the 2021 season due to COVID-19 lockdown interruptions.

**Table 1:** Currie Cup tournament formats and competition’s time of year from 2014 to 2024.

Year	Tournament format	Time of year
**2014**	Double round	Second half of the year
**2015**	Double round	Second half of the year
**2016**	Single round	Second half of the year
**2017**	Double round	Second half of the year
**2018**	Single round	Second half of the year
**2019**	Single round	Second half of the year
**2020/21**	Single round	End of year, and beginning of next year
**2021**	Double round	Second half of the year
**2022**	Double round	First half of the year
**2023**	Double round	First half of the year
**2024**	Single round	Second half of the year

**Table 2:** Injury Incidence, Severity (days), Injury Burden (days absent/1000 player hours) and Operational Burden (days absent due to injuries/match) of Time-Loss injuries for each participating team in the Currie Cup 2024.

Team	Team Injuries / match	Injury Incidence (per 1000 player hours)	Team matches / injury	Total Severity (days lost)	Average Severity (days lost/injury)	Injury Burden (days lost / 1000 hours)	Operational Injury Burden (days lost due to injuries / match)	Median Severity (IQR)
HOLLYWOODbets Sharks	3.1	154.2	0.3	939	25	3913	78.3	17 (12 to 26)
Airlink Pumas	0.7	35.0	1.4	240	34	1200	24.0	41 (31 to 51)
Vodacom Blue Bulls	1.6	81.8	0.6	517	29	2349	47.0	21 (10 to 44)
Toyota Free State Cheetahs	0.9	45.5	1.1	269	27	1224	24.5	23 (15 to 38)
Fidelity ADT Lions	1.8	91.7	0.6	654	30	2726	54.5	29 (12 to 44)
DHL Western Province	2.0	100.0	0.5	557	28	2785	55.7	22 (12 to 51)
Suzuki Griquas	1.3	65.0	0.8	185	14	925	18.5	15 (14 to 20)
Novavit Griffons	0.4	20.0	2.5	266	67	1330	26.6	74 (61 to 80)
***Overall***	***1.5***	***76.2***	***0.7***	***3627***	***28***	***2134***	***42.7***	***21(12 to 43)***

**Table 3:** The Currie Cup 2024 injuries grouped according to the IOC recommended categories of Tissue and Pathology types for injuries.

Tissue	Incidence	Median time loss	Burden
*Pathology*	Injuries per 1000 hours (95%CI)	Days (95%CI)	*Days per 1000 hours (95%CI)*

**Muscle/Tendon**	**16 (10 to 22)**	**17 (0 to 35)**	**592 (370 to 814)**
Muscle Injury	14 (8 to 20)	17 (7 to 27)	420 (240 to 600)
Tendinopathy	2 (0 to 4)	127 (56 to 310)	254 (0 to 508)
**Ligament/Joint capsule**	**26 (18 to 33)**	**41 (33 to 49)**	**1092 (756 to 1386)**
Ligament Sprain	23 (16 to 30)	44 (35 to 53)	989 (688 to 1290)
Joint Sprain	3 (0 to 6)	31 (15 to 47)	87 (0 to 174)
**Nervous**	**19 (12 to 25)**	**13 (10 to 16)**	**323 (204 to 425)**
Brain/Spinal cord injury	17 (11 to 24)	13 (10 to 16)	289 (187 to 408)
Peripheral nerve injury	1 (0 to 3)	20 (5 to 34)*	20 (0 to 60)
**Superficial tissues/skin**	**6 (2 to 9)**	**15 (8 to 22)**	**114 (38 to 171)**
Contusion (superficial)	5 (2 to 9)	15 (7 to 23)	100 (40 to 180)
Laceration	1 (0 to 2)	10	90 (0 to 20)
**Bone**	**8 (3 to 12)**	**38 (26 to 50)**	**320 (120 to 480)**
Fracture	8 (3 to 12)	38 (26 to 50)	320 (120 to 480)
**Cartilage/Synovium/Bursa**	**1 (0 to 2)**	**8**	**72 (0 to 16)**
Bursitis/Synovitis	1 (0 to 2)	8	72 (0 to 16)
**Non-specific**	**2 (0 to 5)**	**13 (7 to 19)**	**22 (0 to 55)**

***Overall***	***76 (63 to 89)***	***21 (16 to 26)***	***2134 (1764 to 2492)***

* The small sample size of peripheral injuries has led to greater variability in the median and 95% confidence intervals.

**Table 4:** Proportion (%) of new versus subsequent recurrent injuries for the Currie Cup 2016 – 2024 tournaments.

	2016	2017	2018	2019	2020/21	2021	2022	2023	2024
New injuries	74	74	86	83	68	71	78	99	97

Subsequent recurrent injuries	2.8	3.2	2.6	2.2	3.4	4.4	3.3	1.3	3.1

**Table 5:** Injury rate, Severity and Burden of the most common injury types in the Currie Cup 2024.

Injury Type	Injury Rate (*95%* CI) *(per 1000 hours)*	Total Severity *(days)*	Average Severity *(days)*	Burden (*95%* CI) *(days lost / 1000 hours)*	Median Severity *(IQR)*
Sprain Ligament	23 (16 to 30)	1326	43	989 (688 to 1290)	44 (21 to 56)
Central Nervous System	17 (11 to 24)	496	17	289 (187 to 408)	13 (12 to 21)
Muscle (Rupture/Strain/Tear)	14 (8 to 20)	715	30	420 (240 to 600)	17 (13 to 46)
Broken Bone/Fracture	8 (3 to 12)	483	40	320 (120 to 480)	38 (27 to 51)
Contusion/Bruise	5 (2 to 9)	176	20	100 (40 to 180)	15 (15 to 28)

***Overall***	***76 (63 to 89)***	***3627***	***28***	***2134 (1764 to 2492)***	***21 (12 to 43)***

**Table 6:** The movement of the most common OSIICS classification diagnoses over the past nine seasons [9]. 2020/21 – was a hybrid tournament structure that started in 2020 and continued into the beginning of the 2021 season due to COVID-19 lockdown interruptions.

			Percentage(%)	Number	Incidence(*95%* CI)	Average Severity
2016		Concussion (HN1)	7	10	6 (2–10)	14
		Knee medial collateral ligamentstrain/tear/rupture (KL3)	6	9	6 (2–10)	23
		Hamstring strain/tear (TM1)	6	8	5 (2–9)	11
2017		Concussion (HN1)	13	16	10 (5–15)	15
		Acromioclavicular joint sprain (SL2)	10	12	8 (3–12)	25
2018		Concussion (HN1)	18	14	15 (7–23)	14
		Quadricep strain (TM2)	5	4	4 (0–8)	18
2019		Concussion (HN1)	12	11	12 (5–18)	9
		Ankle syndesmosis sprain (ALS)	5	5	5 (1–10)	14
2020/21		Concussion (HN1)	8	11	7 (3–11)	10
		Quadriceps haematoma (THQ)	4	6	4 (1–7)	4
		Knee strain (KLZ)	3	5	3 (1–6)	42
2021		Concussion (HN1)	8	11	7 (3–12)	15
		Ankle sprain (ALJ)	6	8	6 (2–10)	10
		Hamstring strain (TM1)	2	5	2 (0–4)	23
2022		Concussion (HN1)	14	17	10 (5–14)	24
		Hamstring strain (TM1)	6	7	4 (1–7)	46
		Ankle syndesmosis sprain (ALS)	4	5	3 (0–5)	12
2023		Concussion (HN1)	28	43	18 (13–24)	16
		Hamstring strain (TM1)	5	7	3 (1–5)	17
		Ankle sprain (ALJ)	3	4	2 (0–3)	19
		Grade 1 medial collateral ligamenttear knee (KLV)	3	4	2 (0–3)	11
2024		Concussion (HN1)	23	30	17 (11–24)	17
		Hamstring strain (TM1)	9	12	7 (3–11)	24
		Ankle syndesmosis sprain (ALS)	6	8	5 (1–8)	60

**Table 7:** Injury rate, Severity and Burden of the most common injured body locations in the Currie Cup 2024.

Injury Location	Injury Rate (*95%* CI) *(per 1000 hours)*	Total Severity *(days)*	Average Severity *(days)*	Burden (*95%* CI) *(*days *lost / 1000 hours)*	Median Severity *(IQR)*
Head	22 (15 to 29)	769	21	462 (315 to 609)	15 (12 to 27)
Ankle	11 (6 to 15)	755	54	594 (324 to 810)	50 (30 to 80)
Knee	10 (5 to 15)	424	30	300 (150 to 450)	28 (12 to 46)
Thigh	10 (5 to 15)	418	25	250 (125 to 375)	15 (13 to 28)
Shoulder	8 (3 to 12)	535	54	432 (162 to 648)	31 (20 to 46)

***Overall***	***76 (63 to 89)***	***3627***	***28***	***2134 (1764 to 2492)***	***21 (12 to 43)***

**Table 8:** Movement of the most injured body locations over the past nine seasons. 2020/21 – was a hybrid tournament structure that started in 2020 and continued into the beginning of the 2021 season due to COVID-19 lockdown interruptions.

			Percentage (%)	Number	Incidence (95% CI)	Average Severity
2016		Knee	14	20	13 (7–18)	49
		Ankle	13	18	12 (6–17)	51
		Head	9	13	8 (4–13)	11
		Shoulder	8	12	8 (3–12)	41
2017		Head	13	16	10 (5–15)	15
		Knee	11	14	9 (4–14)	63
		Shoulder	10	12	8 (3–12)	67
		Ankle	10	12	8 (3–12)	87
		A/C Joint	10	12	8 (3–12)	25
2018		Head	18	14	15 (7–23)	18
		Knee	10	8	9 (3–14)	44
		Shoulder	10	8	9 (3–14)	38
		Ankle	9	7	7 (2–13)	65
		Anterior thigh	8	6	6 (1–12)	6
2019		Head	14	13	14 (6–21)	8
		Knee	13	12	13 (5–20)	13
		Ankle	11	10	10 (4–17)	9
		Lower limb posterior	7	6	6 (1–11)	3
		Posterior thigh	7	6	6 (1–11)	9
2020/21		Head	16	23	14 (9–20)	6
		Knee	15	22	14 (8–19)	57
		Thigh	10	15	9 (5–14)	9
		Shoulder	9	13	8 (4–13)	22
		Ankle	7	10	6 (2–10)	19
2021		Head	15	20	13 (7–19)	9
		Knee	15	20	13 (7–19)	41
		Ankle	14	19	13 (7–18)	13
		Shoulder	11	15	10 (5–15)	18
		Thigh	10	14	9 (4–14)	22
2022		Shoulder	17	21	12 (7–17)	55
		Head	17	20	11 (6–16)	24
		Ankle	15	18	10 (5–15)	30
		Knee	13	16	9 (5–14)	46
		Thigh	13	16	9 (5–14)	29
2023		Head	30	46	20 (14–25)	15
		Knee	20	30	13 (8–17)	34
		Shoulder	11	17	7 (4–11)	25
		Thigh	9	14	6 (3–9)	20
		Ankle	7	10	4 (2–7)	25
2024		Head	29	38	22 (15–29)	21
		Ankle	14	18	11 (6–15)	54
		Thigh	13	17	10 (5–15)	25
		Knee	13	17	10 (5–15)	30
		Shoulder	10	13	8 (3–12)	54

**Table 9:** Injury rate, Severity and Burden of the injury events in the Currie Cup 2024.

*Injury event*	Injury Rate (*95%* CI) *(per 1000 hours)*	Total Severity *(days)*	Average Severity *(days)*	Burden (*95%* CI) *(days lost / 1000 hours)*	Median Severity *(IQR)*
Tackle (Ball Carrier)	22 (15 to 28)	1028	30	660 (450 to 840)	22 (13 to 46)
Open play - contact	16 (10 to 22)	725	29	493 (319 to 667)	28 (15 to 43)
Tackle (Tackler)	15 (9 to 20)	599	29	435 (261 to 580)	17 (12 to 31)
Open play – running	8 (3 to 12)	548	34	280 (102 to 408)	15 (12 to 27)
Ruck	5 (2 to 9)	334	37	185 (74 to 333)	28 (12 to 33)
Maul	2 (0 to 4)	13	13	26 (22 to 52)	13
Scrum	2 (0 to 5)	22	11	22 (0 to 55)	11 (10 to 12)
Kicking	1 (0 to 2)	64	64	64	64
Lineout	2 (0 to 4)	159	53	106 (0 to 212)	50 (37 to 68)
***Overall***	***76 (63 to 89)***	***3627***	***28***	***2134 (1764 to 2492)***	***21 (12 to 43)***

**Table 10:** Injury burden/1000 hours of Time-Loss injuries at the ten Stadia utilised in the Currie Cup combined data from 2016 to 2024.

*Stadium*	*Burden (95%Cl)*
**DHL Cape Town Stadium**	**3668 (2572 to 4765)** §
Danie Craven Stadium	3500 (70 to 7000)
Mbombela Stadium	2945 (2452 to 3438) §
HOLLYWOODbets Kings Park Stadium	2487 (2130 to 2845) §
Emirates Airline Park	2012 (1680 to 2344) *§
Toyota Stadium	1839 (1568 to 2110) *§
Loftus Versveld Stadium	1689 (1421 to 1957) *
Down Touch Investments Stadium	1502 (788 to 2217) *
Midstream College, Olifantsfontein	1425 (29 to 2822)
**Suzuki Stadium**	**1299 (1055 to 1543)** *
***Grouped Average***	***2467 (1976 to 2957)*** §

* Significantly different from DHL Cape Town Stadium

§ Significantly different from Suzuki Stadium

**Table 11:** Injury incidence rate, average-, and median severity of training injuries sustained during the Currie Cup 2024 season according to the type of training activity involved.

	Injury Incidence (*95%CI) (per 1000 player hours)*	Average severity *(days)*	Median severity *(days)*	Burden *(95%CI) Days per 1000 hours*
***Rugby skills (full contact)***	***0.5 (0.2 to 0.8)***	***26***	***14***	***13.0 (5.2 to 21.0)***
Muscle Injury	0.3 (0.0 to 0.5)	23	14	6.9 (0.0 to 11.5)
Ligament Sprain	0.1 (0.0 to 0.3)	5	5	0.5 (0.0 to 1.5)
Joint injury	0.1 (0.0 to 0.2)	3	3	0.3 (0.0 to 0.6)
Tendon Injury	0.1 (0.0 to 0.2)	106	106	10.6 (0.0 to 21.2)
***Rugby skills (semi-contact)***	***1.0 (0.5 to 1.5)***	***24***	***20***	***24.0 (12.0 to 36.0)***
Muscle Injury	0.3 (0.1 to 0.6)	37	34	11.1 (3.7 to 22.2)
Concussion	0.2 (0.0 to 0.4)	21	21	4.2 (0.0 to 8.4)
Unspecified	0.2 (0.0 to 0.4)	26	26	5.2 (0.0 to 10.4)
Ligament Sprain	0.1 (0.0 to 0.3)	15	15	1.5 (0.0 to 4.5)
Bruising/Haematoma	0.1 (0.0 to 0.2)	7	7	0.7 (0.0 to 1.4)
Fracture	0.1 (0.0 to 0.2)	34	34	3.4 (0.0 to 6.8)
Joint Injury	0.1 (0.0 to 0.3)	5	5	0.5 (0.0 to 1.5)
***Rugby skills (non-contact)***	***0.5 (0.2 to 0.8)***	***30***	***21***	***15.0 (6.0 to 24.0)***
Muscle Injury	0.3 (0.0 to 0.5)	18	17	5.4 (0.0 to 9.0)
Ligament Sprain	0.2 (0.0 to 0.4)	50	72	10.0 (0.0 to 20.0)
Tendon Injury	0.1 (0.0 to 0.2)	31	31	3.1 (0.0 to 6.2)
***Weights conditioning***	***0.3 (0.0 to 0.5)***	***37***	***32***	***11.1 (0.0 to 18.5)***
Muscle Injury	0.2 (0.0 to 0.4)	21	24	*4.2 (0.0 to 8.4)*
Joint Injury	0.1 (0.0 to 0.2)	38	38	*3.8 (0.0 to 7.6)*
Unspecified	0.1 (0.0 to 0.2)	84	84	*8.4 (0.0 to 16.8)*
***Non-weights conditioning***	***0.1 (0.0 to 0.3)***	***32***	***32***	***3.2 (0.0 to 9.6)***
Muscle Injury	0.1 (0.0 to 0.2)	53	53	*5.3 (0.0 to 10.6)*
Unspecified	0.1 (0.0 to 0.2)	11	11	*1.1 (0.0 to 2.2)*
***Overall***	***2.4 (1.7 to 3.2)***	***28***	***19***	***67.2 (47.6 to 89.6)***

**Table 12:** Injury incidence rate, average-, and median severity of training injuries sustained per body location, during the Currie Cup 2024.

	Injury Incidence (*95%CI) (per 1000 player hours)*	Average severity *(days)*	Median severity *(days)*	Burden *(95%CI) Days per 1000 hours*
***Head***	***0.2 (0.0 to 0.4)***	***21***	***21***	***4.2 (0.0 to 8.4)***
***Neck***	***0.1 (0.0 to 0.2)***	***11***	***11***	***1.1 (0.0 to 2.2)***
***Upper Body***	***0.3 (0.0 to 0.5)***	***47***	***34***	***14.1 (0.0 to 23.5)***
Wrist/Hand	0.2 (0.0 to 0.4)	15	9	3.0 (0.0 to 6.0)
Shoulder	0.1 (0.0 to 0.2)	84	84	8.4 (0.0 to 16.8)
Elbow	0.1 (0.0 to 0.2)	106	106	10.6 (0.0 to 21.2)
***Lower Body***	***1.7 (1.1 to 2.3)***	***28***	***21***	***47.6 (30.8 to 64.4)***
Thigh	0.7 (0.3 to 1.1)	33	29	23.1 (9.9 to 36.3)
Lower Leg	0.3 (0.1 to 0.6)	27	21	8.1 (2.7 to 16.2)
Knee	0.3 (0.0 to 0.5)	25	7	7.5 (0.0 to 12.5)
Ankle	0.2 (0.0 to 0.4)	28	16	5.6 (0.0 to 11.2)
Hip/Groin	0.2 (0.0 to 0.4)	15	11	7.5 (0.0 to 6.0)
***Trunk***	***0.2 (0.0 to 0.4)***	***5***	***5***	***1.0 (0.0 to 2.0)***
Lumbar Spine	0.2 (0.0 to 0.4)	3	3	0.6 (0.0 to 1.2)
Buttock/Pelvis	0.1 (0.0 to 0.2)	6	6	0.6 (0.0 to 1.2)
Chest	0.1 (0.0 to 0.2)	5	5	0.5 (0.0 to 1.0)
***Overall***	***2.4 (1.7 to 3.2)***	***28***	***19***	***67.2 (47.6 to 89.6)***
